# Myoferlin is a novel exosomal protein and functional regulator of cancer-derived exosomes

**DOI:** 10.18632/oncotarget.13276

**Published:** 2016-11-10

**Authors:** Arnaud Blomme, Karim Fahmy, Olivier Peulen, Brunella Costanza, Marie Fontaine, Ingrid Struman, Dominique Baiwir, Edwin de Pauw, Marc Thiry, Akeila Bellahcène, Vincent Castronovo, Andrei Turtoi

**Affiliations:** ^1^ Metastasis Research Laboratory, GIGA Cancer, University of Liège, Liège, 4000, Belgium; ^2^ Molecular Angiogenesis Laboratory, GIGA Research, University of Liège, Liège, 4000, Belgium; ^3^ Laboratory of Mass Spectrometry, GIGA Research, University of Liège, Liège, 4000, Belgium; ^4^ GIGA Proteomics Facility, University of Liège, Liège, 4000, Belgium; ^5^ Laboratory of Cell Biology, Faculty of Sciences, University of Liège, Liège, 4000, Belgium

**Keywords:** proteomics, vesicle trafficking, angiogenesis, endothelial cells

## Abstract

Exosomes are communication mediators participating in the intercellular exchange of proteins, metabolites and nucleic acids. Recent studies have demonstrated that exosomes are characterized by a unique proteomic composition that is distinct from the cellular one. The mechanisms responsible for determining the proteome content of the exosomes remain however obscure. In the current study we employ ultrastructural approach to validate a novel exosomal protein myoferlin. This is a multiple C2-domain containing protein, known for its conserved physiological function in endocytosis and vesicle fusion biology. Emerging studies demonstrate that myoferlin is frequently overexpressed in cancer, where it promotes cancer cell migration and invasion. Our data expand these findings by showing that myoferlin is a general component of cancer cell derived exosomes from different breast and pancreatic cancer cell lines. Using proteomic analysis, we demonstrate for the first time that myoferlin depletion in cancer cells leads to a significantly modulated exosomal protein load. Such myoferlin-depleted exosomes were also functionally deficient as shown by their reduced capacity to transfer nucleic acids to human endothelial cells (HUVEC). Beyond this, myoferlin-depleted cancer exosomes also had a significantly reduced ability to induce migration and proliferation of HUVEC. The present study highlights myoferlin as a new functional player in exosome biology, calling for novel strategies to target this emerging oncogene in human cancer.

## INTRODUCTION

Exosomes are small extracellular vesicles, ranging in size from 50 to 150 nm. They are derived from endosomal vesicles and secreted into the extracellular medium upon fusion with the plasma membrane [1]. Exosomes assume multiple functions in the context of cancer progression [2]. They notably take part in preparing the metastatic niche [3], have been demonstrated to transform non-tumorigenic cells [4–6], modulate cancer cell metabolism [7] and fuel angiogenesis [8]. Exosome accessibility in liquid biopsies (blood, urine, lymph) and their correlation with patient clinical features (metastatic dissemination, tumor relapse or response to therapy), makes them ideal candidates for diagnostic, prognostic and therapy response markers [9–11]. Recent advances in the field have demonstrated that exosomes are not merely transport containers for intercellular trafficking. They are fully functional entities that contain all the machinery required for *de novo* protein synthesis and processing [12]. These findings are the evidence for the existence of a specific proteome that is confined to exosomes. The understanding of key factors that shape the proteome of exosomes is essential for identifying novel mechanisms that contribute to tumor progression [13, 14].

Myoferlin is a 230kDa trans-membrane multi C2-domain protein that belongs to the ferlin family of proteins. It had been first identified in muscle cells, where it contributes to cell/cell fusion and muscle regeneration [15, 16]. Further studies performed in endothelial cells demonstrated that myoferlin is important for membrane repair and endocytosis [17] as well as receptor-mediated angiogenesis [18–21]. In cancer, overexpression of the protein has been reported in breast, lung, and pancreatic tumors [22–25], where it is associated with increased tumorigenic potential and angiogenesis [21, 26–28]. Mechanistically, myoferlin has been shown to control both endocytosis (EGFR, VEGFR2, IGFR and Tie-2) and exocytosis (VEGF) of several key molecules [18–21, 28]. Inspired by myoferlins' role in cell membrane biology, we hypothesized that myoferlin could be essential to exosomes in regulating their maturation, secretion or internalization. Indeed, our literature research strengthens this hypothesis in showing that myoferlin was found in proteomic analyses of exosomes isolated from different cell lines. Using immunoblotting and electron microscopy, we confirmed that myoferlin is indeed present in exosomes derived from breast and pancreas cancer cells. We showed for the first time that myoferlin is a determining factor for the proteomic diversity of the exosomes, having direct impact on their function. Collectively, our findings place myoferlin on the short list of key proteins important for cancer exosome biology.

## RESULTS

### Myoferlin is expressed in exosomes derived from breast and pancreatic cancer cell lines

**Figure 1:**
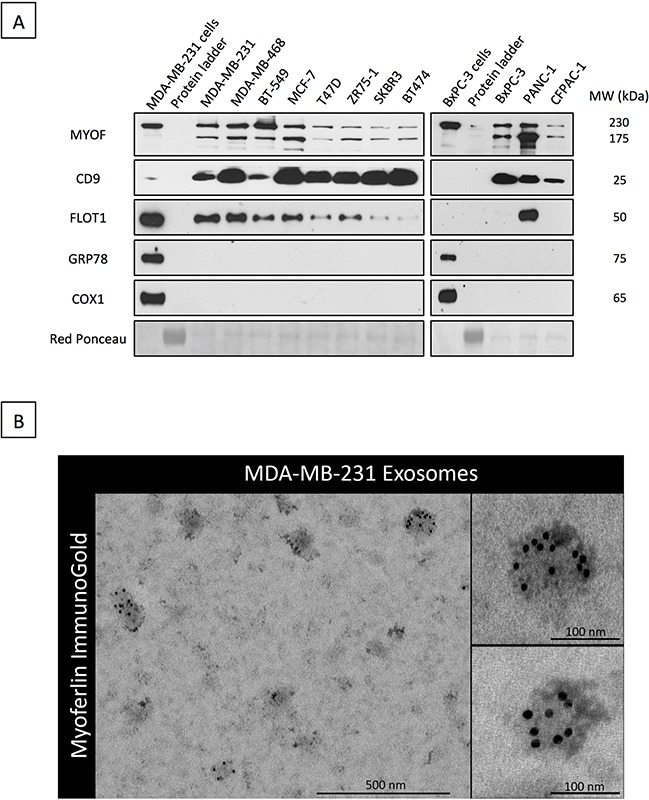
Myoferlin is expressed in exosomes from breast and pancreas cancer cell lines **A.** Western blot analysis of myoferlin expression in exosomes isolated from multiple breast and pancreas cancer cell lines. CD9 and FLOT1 were used as positive controls for exosomes whereas the GRP78 (ER) and COX1 (mitochondria) were employed as controls to exclude presence of contaminating organelles. **B.** Immunogold electron microscopy of exosome preparations from MDA-MB-231 cells stained for myoferlin. (A-B) Representative images of three independent experiments are shown.

In order to investigate the possibility that myoferlin is a constitutive part of exosomes, we first sought to explore existing proteomic data from purified exosomes. Thus, we interrogated the “Exocarta” database (www.exocarta.org) for the presence of myoferlin in extracellular vesicles. Interestingly, we found that myoferlin had been reported in several proteomic analyses of purified exosomes from different cellular origins (Table 1, adapted from exocarta). To validate these *in silico* data, we purified exosomes from the supernatant of breast and pancreatic cancer cell lines, and evaluated myoferlin expression using Western blot. As shown on Figure 1A, the results confirmed the presence of myoferlin in exosomes purified from all the cancer cell lines tested. Interestingly, exosome protein extracts displayed two myoferlin isoforms (~175 kDa and ~230 kDa), while intact cells had predominantly one protein isoform (~230 kDa). It is known that myoferlin gene can undergo alternative splicing (www.uniprot.org). Thus, the ~175 kDa band corresponds to isoform 5, while the ~230 kDa form corresponds to the canonical isoform.

**Table 1:** Myoferlin expression in cancer-derived exosomes, adapted from exocarta (http://www.exocarta.org/)

Gene Name	Analysis	Origin	Authors	Journal	Year
MYOF	Proteomic analysis of exosomes	Bladder Cancer Cells	Welton, et al.	Mol. Cell. Proteomics	2010
MYOF	Proteomic analysis of exosomes	Colon Cancer Cells	Mathivanan, et al.	Mol. Cell. Proteomics	2010
MYOF	Proteomic analysis of exosomes	Colon Cancer Cells	Demory Beckler, et al.	Mol. Cell. Proteomics	2013
MYOF	Proteomic analysis of exosomes	Hepatocellular Carcinoma Cells	He, et al.	Carcinogenesis	2015
MYOF	Proteomic analysis of exosomes	Melanoma Cells	Lazar, et al.	Pigment Cell. Melanoma Res.	2015
MYOF	Proteomic analysis of exosomes	Ovarian Cancer Cells	Liang, et al.	J. Proteomics	2013
MYOF	Proteomic analysis of exosomes	Prostate Cancer Cells	Kharazia, et al.	Oncotarget	2015
MYOF	Proteomic analysis of exosomes	Squamous Carcinoma Cells	Park, et al.	Mol. Cell. Proteomics	2010
MYOF	Proteomic analysis of exosomes	Urine	Gonzales, et al.	J. Am. Soc. Nephrol.	2009

In the present work, the assessment of exosome purity was conducted using CD9 and FLOT1 exosome markers. GRP78 (endoplasmic reticulum) and COX1 (mitochondria) were employed as controls to exclude presence of contaminating organelles. The quality of exosome preparation was further verified using DLS (particle size distribution) and electron microscopy (size and shape) (Supplementary Figure S1, Supplementary Data). The presence of myoferlin in cancer-derived exosomes was verified by electron microscopy. This was done by using an anti-myoferlin immunogold labelling of purified exosomes from MDA-MB-231 breast cancer cells (Figure 1B). Following these findings, we next examined if myoferlin silencing had an impact on exosome structure and content with the goal of elucidating the potential role of myoferlin in exosomes.

### Myoferlin silencing affects the size and the protein content of tumor-derived exosomes

**Figure 2:**
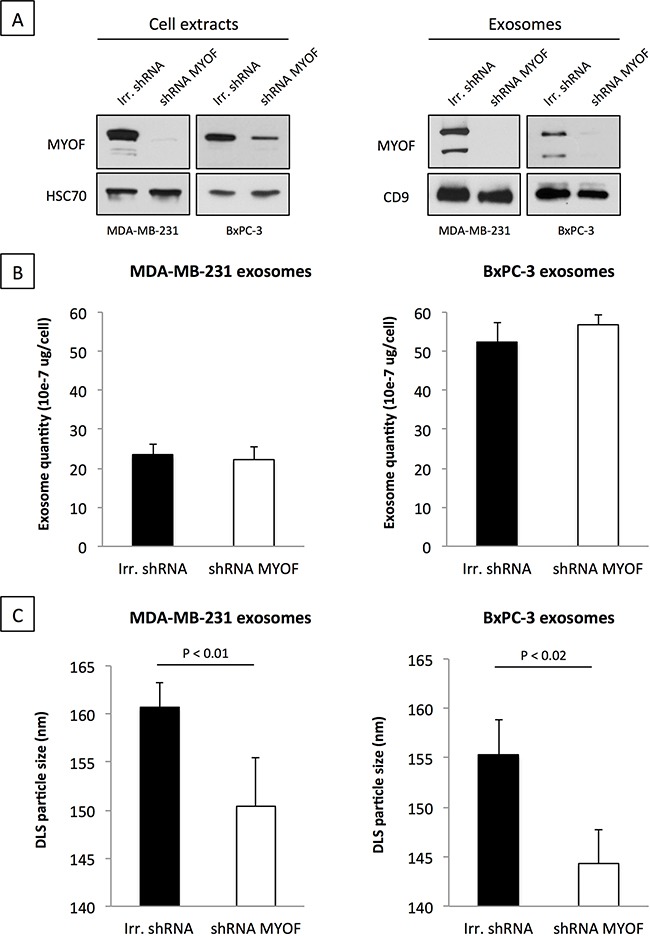
Loss of myoferlin decreases exosome size but not quantity **A.** Western blot validation of myoferlin depletion in cells (left) and exosome preparation (right) purified from breast (MDA-MB-231) and pancreas (BxPC-3) cancer cell supernatants. HSC70 (cell extracts) and CD9 (isolated exosomes) were used as loading controls. Representative images of three independent experiments are shown. **B.** Exosome quantity was assessed by protein quantification of the total amount of isolated exosomes relative to the number of cells. **C.** Measurement of average exosome size using DLS. (B-C) Data are averages of three independent experiments; error bars indicate standard error of means.

**Figure 3:**
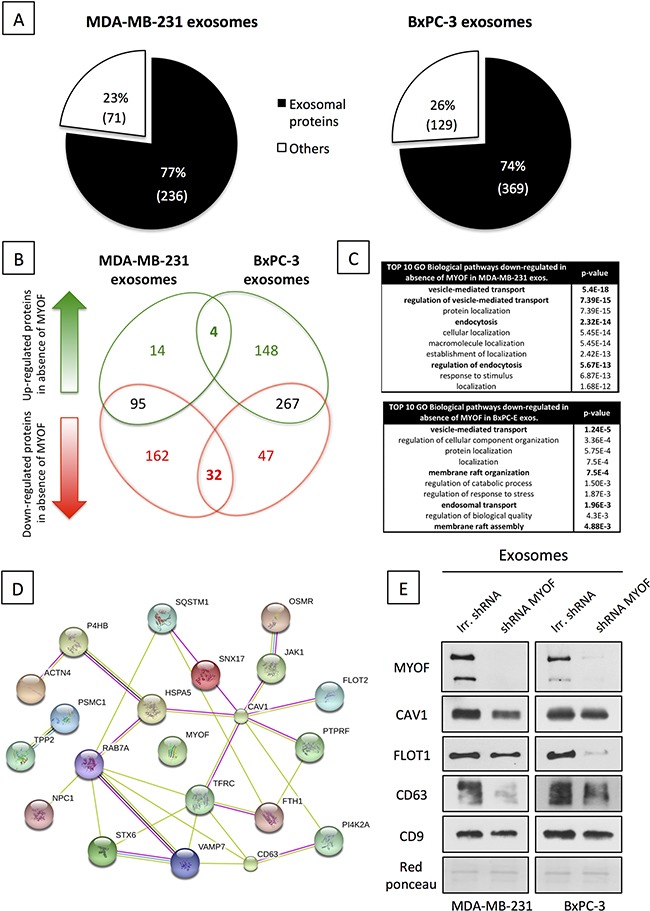
Myoferlin depletion induces major change in cancer exosome proteome content **A.** Exosomal localization of proteins identified in MDA-MB-231 (left) and BxPC-3 (right) proteomic analysis of isolated exosomes, according to the STRING database (http://string-db.org/). **B.** Venn diagram showing the differential repartition of modulated proteins in MDA-MB-231 and BxPC-3 isolated exosomes. (A-B) The values are averages of two independent experiments. **C.** Enrichment pathway analysis of down-regulated proteins in absence of myoferlin. **D.** STRING Protein interaction network of proteins commonly down-regulated in breast and pancreatic myoferlin-deficient exosomes **E.** Western blot validation of vesicular makers modulated in response to myoferlin silencing (CD9 was used as control). Red Ponceau is used as a loading control. Shown are representative images of three independent experiments.

As a first step towards characterizing myoferlin-deficient exosomes we sought to assess the modulation of their quantity and size in MDA-MB-231 and BxPC-3 cancer cells. Myoferlin silencing in both MDA-MB-231 and BxPC-3 cells efficiently reduced myoferlin load in the cancer exosomes (Figure 2A). Exosome quantity was estimated by determining their total protein content. As shown in Figure 2B, no major difference in the quantity of exosomes, produced by myoferlin-depleted cells, was found in comparison to the control condition. However, using DLS, we observed that exosomes derived from myoferlin-depleted cells showed a significantly smaller size (Figure 2C). To further investigate this observation, we tried to understand if the proteome identity of myoferlin-depleted exosomes is different from those that kept myoferlin. Therefore, we performed a proteomic analysis of exosomes isolated from MDA-MB-231 and BxPC-3 cells that were silenced for myoferlin, and compared them to control exosomes. We identified 307 and 498 proteins in MDA-MB-231 and BxPC-3 conditions respectively (Supplementary Table S1). Expectedly, over 75% of the identified proteins were predicted to have an exosomal subcellular localization (Figure 3A). Although the identity of the modulated proteins was different between breast and pancreatic exosomes (only 36 proteins overlapped, Figure 3B, Supplementary Table S2 and Supplementary Table S3), we observed that the regulatory pathways down-regulated in absence of myoferlin were highly similar (Figure 3C). Indeed, in both cell types, these pathways were related to endocytosis and other vesicle-mediated processes. The majority of the commonly modulated proteins were decreased in myoferlin-depleted exosomes (32 out of 36 proteins, Figure 3C). Thus, we focused on down-regulated proteins that were observed in exosomes from both cells lines, and investigated if these proteins belong to a functional network. To achieve this, we employed publicly available software STRING (Figure 3D). Of 32 down-regulated proteins, 18 were participating in a network. Noteworthy were the proteins essential for proper intracellular vesicle transport (CAV1, FLOT1, FLOT2, SXN6) and key regulators of the endo-lysosomal trafficking (VAMP7, RAB7A), both processes requiring high number of vesicle/membrane fusion events. Interestingly, we also observed a consistent decrease in CD63 protein expression, which is an important marker of extracellular vesicles. Decreased expression of several modulated proteins (CAV1, FLOT1 and CD63) was further validated using Western blot on purified exosome extracts (Figure 3E). Following myoferlin-depletion, our data established a significant modulation of many exosome-specific proteins. This prompted us to ask the question if such altered exosomes possibly have an impaired function. Considering their essential role in cell-to-cell communication, we next sought to assess if cancer-derived and myoferlin-depleted exosomes would have a differential impact on human endothelial cells.

### Myoferlin silencing alters exosome fusion and cargo delivery in target cells

**Figure 4:**
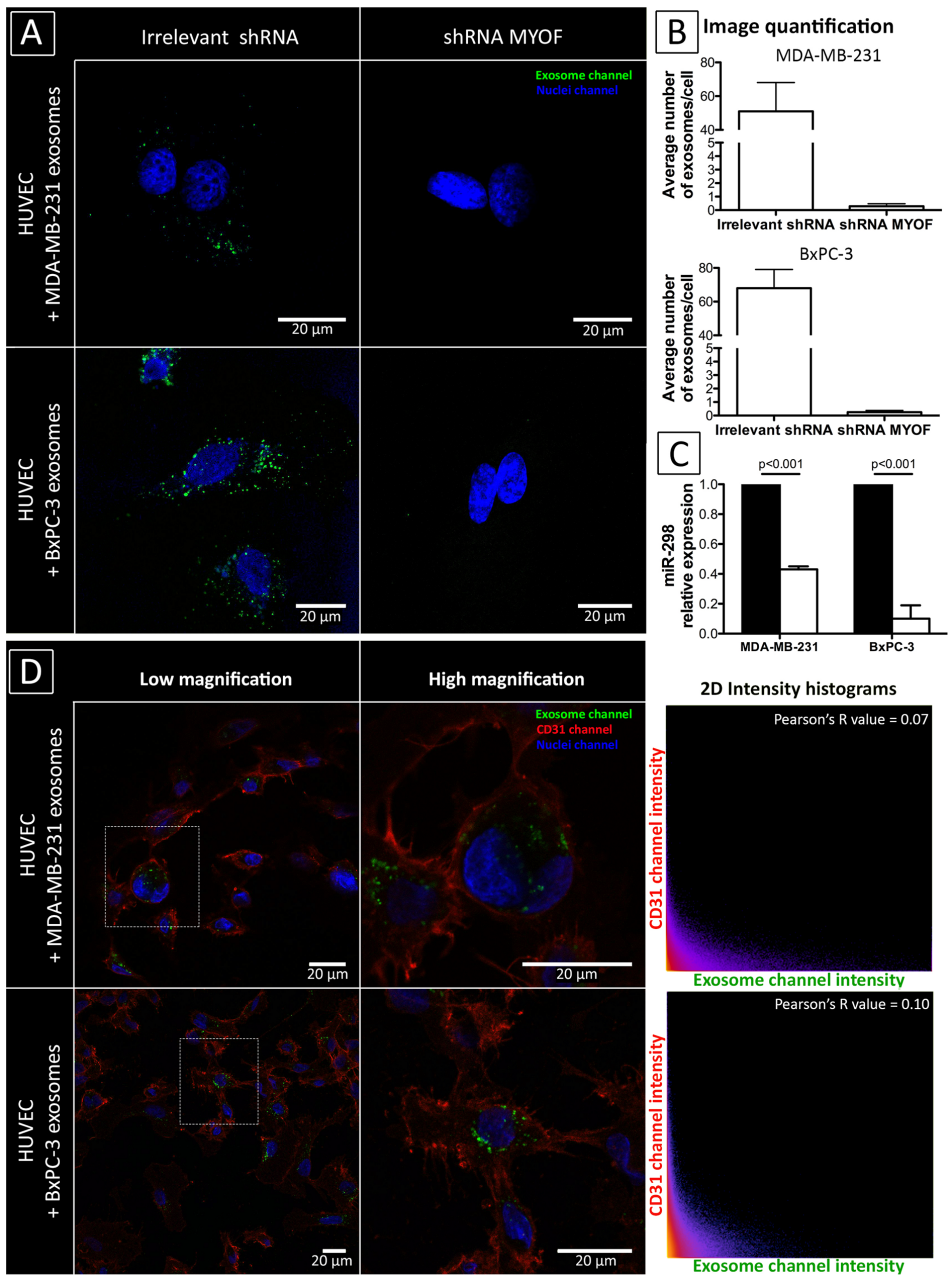
Myoferlin-deficient exosomes are impaired to enter target cells **A-B.** Immunofluorescence of HUVEC incubated with PKH67 labelled cancer-derived exosomes. Shown are representative images of three independent experiments. Quantification was conducted on 10 randomly selected fields, error bars indicate standard error of means. **C.** Relative levels of the murine miRNA miR-298, measured by qRT-PCR, in HUVEC incubated with cancer-derived exosomes pre-loaded with miR-298. Ubiquitously expressed RNU44 was used for normalization. Data are averages of three independent experiments; error bars indicate standard error of means. **D.** Confocal analysis of cancer-derived exosomes localization in HUVEC. CD31 was used for membrane staining while nuclei were labelled with DAPI. The absence of cell surface adsorption was excluded using the Pearson correlation analysis.

In order to further understand the molecular effects of myoferlin-depletion in cancer exosomes, we first tested their ability to fuse with human umbilical vein endothelial cells (HUVEC). For this purpose, we treated endothelial cells with PKH67-labelled exosomes, isolated from control and myoferlin-depleted cancer cells. Exosome uptake in HUVEC was visualized using the immunofluorescent labelling. As shown in Figure 4A, endothelial cells treated with control exosomes displayed a strong intracellular signal, confirming the entry of vesicles in the target cells. In contrast to this, we could not detect any fluorescence in HUVEC incubated with exosomes lacking myoferlin. Next, we tested the transfer of specific miRNA from cancer cells to endothelial cells. Accordingly, we transfected a murine miRNA (miR-298) in cancer cells (in both control and myoferlin depleted condition), purified the exosomes from these cells and then incubated them with HUVEC. As shown in Figure 4C and in comparison to control conditions, the transfer of the miR-298 was significantly decreased in HUVEC treated with exosomes lacking myoferlin. In order to exclude the possibility that exosomes are adsorbed on the surface of HUVEC we have stained membrane protein CD31 in HUVEC pre-incubated with PKH67-labelled exosomes (Figure 4D). The Pearson correlation analysis between CD31 and exosome signal, obtained through confocal imaging, confirmed that both do not colocalize and that exosomes are indeed found inside the HUVEC. Having found that myoferlin is essential for the exosomal transfer of molecules between the cells, we next examined if this would translate in biological consequences.

### Myoferlin silencing impairs exosome-mediated reprogramming of endothelial cells in vitro

**Figure 5:**
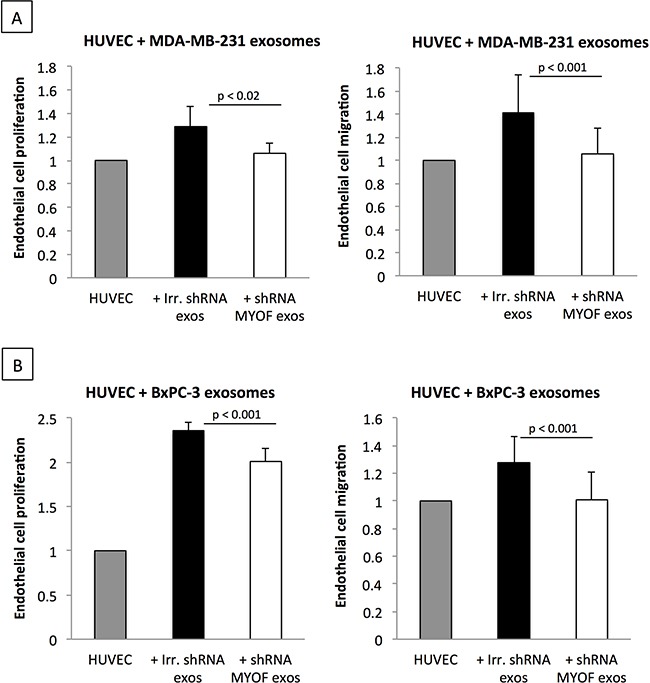
Myoferlin-depleted cancer-derived exosomes decrease proliferation and migration of endothelial cells **A.** Proliferation and **B.** migration of endothelial cells incubated with exosomes isolated from myoferlin-deficient or control cancer cells. (A-B) Data are averages of three independent experiments; error bars indicate standard error of means.

Cancer-derived exosomes are known angiogenic mediators in the complex crosstalk between cancer and endothelial cells [3]. We made use of this known biological mechanism to examine the functional impact of myoferlin-depletion in MDA-MB-231- and BxPC-3-derived exosomes. As outlined in Figure 5A-5B, endothelial cells incubated with control cancer cell-derived exosomes displayed increased rates of proliferation and migration. However, when the HUVEC were incubated with myoferlin-depleted exosomes, these effects were significantly decreased, restoring the proliferation and migration responses similar to those of non-treated cells (Figure 5A-5B).

## DISCUSSION

Cancer cell-stroma communication represents a key mechanism required for both tumor growth and metastatic dissemination. A paucity of studies demonstrates that exosomes are mediators that facilitate intercellular exchange of biomolecules, assuming an important role in cell-to-cell communication [29]. In the current study we focused on myoferlin, a membrane associated protein, which has recently been demonstrated to be pro-oncogenic in several types of cancer [21, 22, 26–28, 30]. In breast cancer, *in vivo* tumor xenografts lacking myoferlin have been described as smaller, less invasive and less vascularized than their control counterparts [27]. Antitumor effects observed following myoferlin depletion have been attributed to impaired membrane repair/turnover [22], the inability to properly internalize growth factor receptors [21], impaired cancer cell motility [21, 27, 30] and the inability to sustain tumor-associated angiogenesis [20, 28]. However, our study brings forward an additional important role for myoferlin in tumor progression, namely its ability to exert functional impact on exosome biology. Indeed, although the presence of exosomal myoferlin has been reported in several proteomic analyses of tumor-derived exosomes (see Table 1), to our knowledge, the definitive proof of its presence in exosomes has not been provided as yet. Strengthened by previous proteomic studies (Table 1), we prove by Western blotting and electron microscopy that myoferlin is indeed a major component of cancer cells-derived exosomes. We further confirm that myoferlin is present in exosomes derived from all major breast and pancreatic cancer cell lines from different genetic backgrounds. To exclude any bias from the isolation method, we have performed alternative exosome purification using sucrose density-gradient ultracentrifugation. The results have confirmed myoferlin presence in the exosomes (Supplementary Figure S2, Supplementary Data). Although the experiments were not quantitatively conducted with the ones described in the Figure 1A, weaker myoferlin bands in Supplementary Figure S2 might suggest that not all extracellular myoferlin is present inside exosomes. At this point we cannot exclude a theoretical possibility that a portion of myoferlin is additionally released in the extracellular space by alternative secretion methods. Further studies are thus warranted to fully investigate the nature of extracellular myoferlin presence.

Interestingly, in the current study we detected myoferlin at two molecular weights, ~230 kDa and ~175 kDa, that corresponded to canonical isoform and isoform 5 respectively. Regardless of the purification method, the cancer cell derived exosomes are particularly enriched for the isoform 5 (Figure 1, Figure 2 and Supplementary Figure S2). Despite their existence, the function of individual myoferlin splice variants is not understood. Therefore we are at present unable to explain what is the respective function of these two protein isoforms and future efforts should concentrate on clarifying their function.

The focus of the current study has been placed on cancer cells, because myoferlin expression has been reported to be particularly high in cancer. However, the question if myoferlin is also present in exosomes derived from normal cells is certainly of broad biological interest. In this context we have performed exosome isolations from two different non-malignant/healthy cells, MCF10A (non-transformed breast epithelial cells) and HUVEC (endothelial cells). Following this we have examined the myoferlin content and have found that the canonical isoform of myoferlin is indeed present in the exosomes derived from both cell lines (Supplementary Figure S3, Supplementary Data). These findings suggested that myoferlin might be present in exosomes of normal cells that contain basal levels of this protein. This is further supported by literature data showing that myoblasts-derived exosomes, which are normal cells known to contain significant amounts of myoferlin, are also rich in myoferlin [31]. Overall, myoferlin appears to be relevant to exosome biology beyond cancer and this calls for further in depth studies that will in particularly clarify the role of myoferlin in exosomes derived from normal cells.

Our initial functional analysis showed that myoferlin depletion in cancer cells does not affect exosome quantity but it does affect their size. This suggested that myoferlin could influence exosome cargo content rather than exosome production by cancer cells. These findings further motivated us to examine the protein content of myoferlin-depleted exosomes and compare this to the control counterparts. The proteomic analysis of isolated exosomes revealed that myoferlin silencing leads to a significant alteration of the protein content. Notable was the decreased expression of several vesicular markers that are known to be expressed in exosomes (caveolin-1, flotilin-1 and 2, syntaxin 6). The analysis also demonstrated the modulation of several members of the RAB family of RAS-related GTP-binding proteins, which are important regulators of vesicular transport. Of these, RAB7A was decreased in myoferlin-depleted condition. RAB7A is a known marker of endo-lysosomal transition whose upregulation has been associated with tumorigenesis in multiple organs [32–34]. Recently, Redpath et al. showed that myoferlin strongly localized in RAB7-positive late endosomes [35]. The decrease of RAB7A expression observed in absence of myoferlin could therefore reflect another mechanism that could explain the observed anti-tumor effect. A further important observation resulting from the current proteomic study was the marked decrease of CD63 following myoferlin silencing. CD63 is a widely used exosomal marker and its expression correlates with increased aggressiveness and metastatic potential of breast tumors [36, 37]. Our data also pointed to reduced levels of ferritin and transferrin levels in myoferlin-depleted exosomes, suggesting that myoferlin could also be an important factor regulating iron metabolism. Iron metabolism is frequently deregulated in cancer [38] and high levels of serum ferritin have been shown to correlate with adenoma formation in colon [39], poor clinical outcome of pancreatic cancer patients [40] and response to chemotherapy in breast cancer [41, 42].

Exosomes are thought to support tumor growth by reprogramming stromal cells such as fibroblasts and endothelial cells [3, 43]. In particular, several studies have demonstrated the ability of cancer-derived exosomes to induce angiogenesis [44–46]. Of several critical steps, the entry of exosomes into target cells, which involves also a direct fusion with the plasma membrane [1], is an important event in the exosome-mediated signalling. The present study demonstrates for the first time that silencing myoferlin impedes exosome uptake by endothelial cells. This, in addition to the altered protein content, leads to diminished ability of myoferlin-depleted exosomes to stimulate endothelial cell migration and proliferation. Although the consequences for tumor progression are evident, additional studies need to be conducted in order to understand how myoferlin contributes to the exosome uptake in the target cells. Hints towards answering this question may be provided by the recent study of Melo et al. [12] who elegantly demonstrated that an exosomal maturation process outside the cell is required to achieve proper release of their proteic and nucleotidic content in the target cells and subsequently initiate tumorigenesis. The fact that myoferlin is able to determine protein cargo of cancer-derived exosome could explain why this protein is overexpressed in cancer, and further underlines its importance in the regulation of both intra- and extracellular vesicle trafficking of cancer cells.

## MATERIALS AND METHODS

### Cell culture and myoferlin-depleted cell lines

The cell lines used in this study (MDA-MB-231 (HTB-26), MDA-MB-468 (HTB-132), BT-549 (HTB-122), Hs 578T (HTB-126), MCF7 (HTB-22), MCF-10A (CRL10317), ZR-75-1 (CRL-1500), BT-474 (HTB-20), SK-BR-3 (HTB-30) and CFPAC-1 (CRL-1918)) were obtained from American Type Culture Collection (ATCC; Manassas, VA, USA). Human pancreatic adenocarcinoma cells BxPC-3 (ATCC CRL-1687) were a generous gift from Prof. Bikfalvi (Inserm U1029, Bordeaux, France). PANC-1 cells (ATCC CRL-1469) were a generous gift from Prof. Muller and Burtea (NMR Laboratory, University of Mons, Belgium). All cells were cultured in their recommended medium at 37°C, 5% CO_2_, and 95% humidity. Isolation of human umbilical vein endothelial cells (HUVEC) (passages 6–11) was described previously [47]. HUVEC were seeded onto gelatin-coated culture flasks, cultured in EGM-2 medium (Cat. #: CC3162/6; Lonza, Basel, Switzerland) and supplemented with 5% DBS (Cat. #: 10371029; Gibco-Thermo Fisher).

Stable myoferlin depletion was achieved using lentiviral shRNA particles. Myoferlin shRNA plasmid (sequence CCCUGUCUGGAAUGAGA cloned into pLKO, Sigma, St. Louis, MO, USA) or control shRNA (Cat. #: SHC005; Sigma, St. Louis, MO, USA) were cotransfected with pSPAX2 (Addgene, Cambridge, MA, USA) and a VSV-G encoding plasmids [48] in lenti-X 293T cells (Clontech, Mountain view, CA, USA). Titration of the lentiviral vectors was performed using the qPCR Lentivirus Titration (Titer) Kit (Cat. #: LV900; ABM, Richmond, BC, Canada). After transduction, the stably transduced clones were selected following Puromycin treatment (10 μg/mL, ant-pr-1, Invivogen, Toulouse, FR).

### Exosome purification

Cancer cells were seeded at 75% confluence and allowed to grow in their respective culture medium supplemented with exosome-depleted serum. After 48 h, supernatant was first centrifuged for 20 minutes at 2,000g to remove floating cells. Cell-free supernatant was next centrifuged at 12,000g for 45 minutes and filtered (0.22 μm filter) to remove additional cell debris. The collected medium was then ultracentrifuged for 2 h at 100,000g using SW-28 rotor and Optima LE80 ultracentrifuge (Beckman Coulter, Brea, CA, USA). The exosome containing pellet was washed once with PBS, centrifuged again for 2 h at 100,000g and finally suspended either in PBS or SDS-based lysis buffer (50 mM Tris-HCl, 1% SDS, pH 7.5, supplemented with protease and phosphatase inhibitors (Cat. #: 11873580001; Roche, Mannheim, Germany). The PBS fraction was used for functional assays and microscopy, whereas the SDS-lysate for Western blot and proteomic analysis. All centrifugation steps were conducted at 4°C.

### Exosome quantification and purity assessment

Exosome quantification was performed from the SDS lysates (see above) using BCA protein assay kit (Cat. #: 23225, Thermo Scientific). The protein quantity was normalized to the number of cells that was used to isolate the exosomes. Exosome size distribution was assessed using dynamic light scattering (DLS) methodology. For this purpose exosomes were suspended in PBS at a concentration of 100 μg/mL and the analysis was performed with Zetasizer Nano ZS (Malvern Instruments, Ltd., Worcestershire, UK) DLS instrument. Intensity, volume and distribution data for each sample were collected on a continuous basis for 4 minutes in sets of 3.

### Electron microscopy and immunogold labeling of isolated exosomes

Labeling of cell-derived exosomes was performed as previously described [47]. Briefly, exosomes attached on formvar-carbon coated grids were successively washed, fixed in 2% formaldehyde and incubated with primary (2 h, RT, anti-MYOF (Cat. #: HPA014245; Sigma Aldrich, St. Louis, MO, USA) 1/20 dilution in PBS-BSA 0.2% supplemented with normal goat serum 1/50) and secondary antibodies (1 h, RT, anti-rabbit coupled with gold particles (Aurion, Wageningen, The Netherlands) diluted 1/40 in PBS-BSA 0.2%, pH 8.2) for 1 h. Samples were postfixed for 10 minutes in 2.5% glutaraldehyde and counterstained using uranyl acetate and lead citrate. Pictures were made with a Jeol JEM-1400 transmission electron microscope (TEM) at 80 kV (Jeol, Peabody, MA, USA). When immunogold labeling was not required (structural observations), exosome preparation for TEM commenced with fixation step.

### Western blotting

Cells or exosome pellets were first lysed in SDS-based buffer. Protein quantification was performed using the BCA quantification kit (Pierce, Thermo Scientific, Rockford, IL, USA). Desired amount of proteins were mixed with Laemmli buffer (60 mM Tris-HCl pH 6.8, 25% glycerol, 2% SDS, 14.4 mM of 2-mercaptoethanol and trace of bromophenol blue) and denatured for 5 minutes at 99°C. Protein samples were separated by SDS-PAGE followed by electro-transfer on PVDF membrane. The membranes were incubated with the selected primary antibodies (outlined below) overnight at 4°C and then probed with corresponding secondary antibody conjugated to horseradish peroxidase (anti-rabbit antibody (Cat. #: G21234; Life-Technologies, Gent, BE) and anti-mouse antibody (Cat. #: P0260; Dako, Glostrup, Denmark) for 1 h at room temperature. The immunoblots were revealed using the chemiluminescent substrate (ECL Western blotting substrate, Thermo Scientific, Rockford, IL, USA). Following antibodies were used: myoferlin (Cat. #: HPA014245; Sigma Aldrich, St. Louis, MO, USA), caveolin-1 (Cat. #: 3238; Cell Signaling, Danvers, MA, USA), flotilin-1 (Cat. #: 3253; Cell Signaling), CD-9 (Cat. #: 2125-0909; AbD Serotec, Düsseldorf, Germany), CD-63 (Cat. #: ab134045; Abcam, Cambridge, United Kingdom) and HSC70 (Cat. #: sc-7298; Santa Cruz, Dallas, TX, USA).

### Proteomic analysis of cancer cell-derived exosomes

Exosome purifications were performed in biological duplicates. Exosome pellets were directly lysed in the SDS-based lysis buffer supplemented with protease and phosphatase inhibitors (for details see above). Protein extracts were further reduced with dithiothreitol (100 mM), alkylated with 150 mM of chloroacetamide and precipitated with 20% trichloroacetic acid. Protein pellets were then washed with acetone, suspended in ammonium bicarbonate buffer (50 mM NH_4_HCO_3_, pH 8.0) and digested with trypsin (sequencing grade, 1/50: protease/protein; Promega, Madison, WI, USA). Finally, 5 μg of peptides were desalted using C18 ZipTip (Millipore, Billerica, MA) and 1.5 μg were injected on an Acquity M-Class UPLC (Waters, Milford, MA, USA) connected to a Q Exactive Plus (Thermo Scientific, Bremen, Germany), in nano-electrospray positive ion mode. The samples were loaded on the trap column (Symmetry C18 5μm, 180 μm x 20 mm, Waters) in 100% solvent A (water 0.1% formic acid) during 3 minutes and subsequently separated on the analytical column (HSS T3 C18 1.8 μm, 75 μm x 250 mm, Waters); flow rate 600 nL/min, solvent A (0.1% formic acid in water) and solvent B (0.1% formic acid in acetonitrile), linear gradient 0 min, 98% A; 5 min, 93% A; 135 min, 70% A; 150 min, 60% A, total run time was 180 min. The MS acquisition was conducted in data-dependent mode. The parameters for MS acquisition were: MS range from 400 to 1750 *m/z*, Resolution of 70,000, AGC target of 1e^6^ or maximum injection time of 50 ms. The parameters for MS2 spectrum acquisition were: isolation window of 2.0 *m/z*, normalized collision energy (NCE) of 25, resolution of 17500, AGC target of 1e^5^ or maximum injection time of 50 ms, underfill ratio of 1.0%. Protein identifications and quantifications were conducted using MaxQuant v1.5.2.8 using UniProt® human database. Normalization of the data was done using LFQ algorithm [49]. Peptide modification carbamidomethylation (C) was set as fixed and oxidation (M) as variable. The minimum ratio count for LFQ was set at 2. The main search tolerance was set at 5 ppm (10 ppm for MS/MS). Peptide spectrum match (PSM) and protein false discovery rates (FDR) were both set at 0.01. Enrichment of GO biological pathways as well as the network analysis among the modulated proteins was conducted using the STRING program (http://string-db.org/) [50].

### Immunofluorescence and quantification

Exosomes isolated from cancer cells were labelled with PKH67 dye (Cat. #: P7333; Sigma Aldrich, Saint-Louis, USA) according to the manufacturer's instructions. Then, fluorescently-labeled exosomes were incubated with endothelial cells (HUVEC, seeded at 30,000 cells in 24-well plate) for 2-4 h. Following this treatment, the cells were fixed for 10 minutes in 4% PFA, washed two times with PBS and blocked for 30 min with PBS/BSA (2%). In order to visualize the plasma membrane, HUVEC were labelled with anti-CD31 (dilution 1:20; Cat. N#: M0823, Dako) for 2h at RT. Following three PBS washes, the samples were incubated with Alexa633 goat-anti-mouse IgG (dilution 1:2000; Cat. #: A21050, Life Technologies) for 45 min. After PBS wash, the slides were mounted (Fluorescent Mounting Medium; Cat. #: S3023, Dako) and visualized under a confocal microscope. Images were acquired using TCS-SP5-A0BS confocal microscope (Leica, Wetzlar, Germany) at a magnification of 63X.

Exosome uptake was quantified using the particle analyzer of Fiji/ImageJ 1.51d [51] after image binarization. Colocalization of uptaken PKH67-labelled exosomes and CD31 was evaluated after confocal Z-stack image acquisition. Split channel images were submitted to a Pearson's correlation analysis using the Fiji/ImageJ 1.51d Coloc 2 plugin.

### miRNA overexpression and exosome-mediated transfer to endothelial cells

Murine specific miRNA miR298 (Cat. #: PM12525; Thermo Scientific, Rockford, IL, USA) was transfected at a concentration of 25 nM in cancer cells using Lipofectamine 2000 reagent (Cat. #: 11668-019; Invitrogen, Carlsbad, California, USA). MiR298-overexpressing exosomes were then isolated and incubated with HUVEC (seeded at 30,000 cells in 24-well plate) for 2 h. Following exosome treatment, total RNA was extracted from the endothelial cells using the DNA, RNA, and protein purification kit - Nucleospin (Cat. #: 740955.250; Macherey-Nagel, Duren, Germany). Reverse transcription (RT) was performed using the RevertAid H Minus First Strand cDNA Synthesis Kit (Cat. #: K1631; Thermo Scientific), replacing the random hexamer primers with miR-298 and RNU44 RT-specific primers (same Cat.# as for PCR-primers described below). Detection of the miR-298 miRNA was then performed using Quantitative PCR, with PCR-specific primers miR-298 (Cat. #: 002598; Applied Biosystems, Foster City, California, USA) and RNU44 (Cat. #: 001094; Applied Biosystems, Foster City, California, USA). RNU44 was used for normalization.

### Proliferation of exosome-treated endothelial cells

HUVEC were plated at 2 × 10^3^ cells/well in 96-well plates and cultured overnight in EGM-2 (100 μl/well). Cells were incubated with 1.5 μg exosomes per well for 72 h in EBM-2 (Cat. #: CC3156; Lonza) with 0.5% DBS. Bromodeoxyuridine (BrdU) was added and the culture was incubated for 8h. BrdU incorporation was measured with the Cell Proliferation ELISA BrdU (chemiluminescence) kit (Roche) according to the manufacturer's protocol.

### Migration of exosome-treated endothelial cells

HUVEC were plated at 5 × 10^4^ cells/well in 48-well plates and cultured overnight in EGM-2 (300 μl/well). Cells were incubated with 5 μg exosomes per well for 24 h. To examine wound healing, the confluent monolayers were mechanically scratched using a pipette tip to create the wound. Cells were washed with PBS and EBM-2 (Cat. #:CC3156; Lonza) 0.5% DBS (Cat. #: 10371029; Gibco-Thermo Fisher) was added to allow for wound healing. The distance between the two sides of the wound was measured with a graduated ocular lens and a microscope (CKX41; Olympus, Tokyo, Japan). The distance between the two sides of the wound after 6h of migration was subtracted from the distance at time 0 and represented on a graph.

### Statistical analysis

Proteomic analysis of exosomes from MDA-MB-231 and BxPC-3 cells was conducted in biological duplicate. All other experiments were carried out in biological triplicates. Statistical analysis was conducted using two-sided Student's t-test and assuming equal variances (Excel; Microsoft, Redmond, WA, USA). The *t*-test was considered appropriate because data followed a normal distribution (Shapiro-Wilk test, threshold 0.05). All error bars indicate standard error of means.

## SUPPLEMENTARY METHODS








