# Supplementary material for: Myoferlin is a novel exosomal protein and functional regulator of cancer-derived exosomes
Source: Oncotarget. 2016 Nov 10;7(50):83669–83. doi: 10.18632/oncotarget.13276 (PMC5347796; doi:10.18632/oncotarget.13276)
Supplement: Supplementary file 1 [file oncotarget-07-83669-s001.pdf]

Myoferlin is a novel exosomal protein and functional regulator of cancer-derived exosomes

SUPPLEMENTARY METHODS

Sucrose density-gradient exosome purification

Cell culture medium was collected, centrifuged to remove cell debris and filtered as described in the main Materials and Methods section. The supernatant was further purified by underlying it with a heavy sucrose cushion (30% sucrose mixed in deuterated water) and subjected to ultracentrifugation at 100.000g for 2 h at 4°C. The cushion was then collected, washed with 30 mL of PBS and ultracentrifuged at 100.000g for 2 h at 4°C. Finally, the exosome pellet was suspended in lysis buffer and was used for Western blotting.

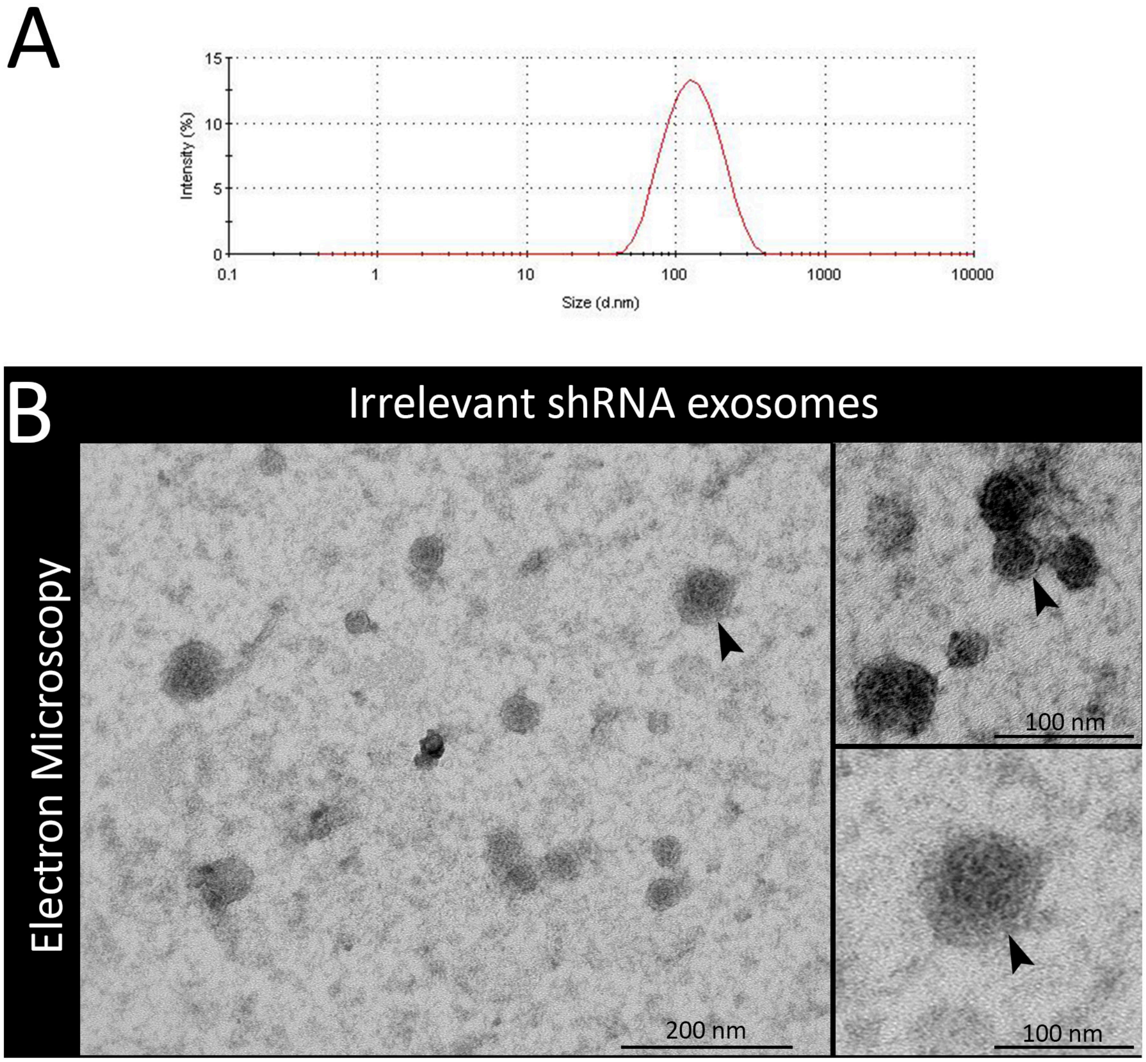

Supplementary Figure S1: Quality control of isolated exosomes. A. The size of exosomes was determined using the DLS analysis. **B.** The purity and morphology was additionally assessed using TEM. (A-B) Representative images of exosomes isolated from BxPC-3 cells.

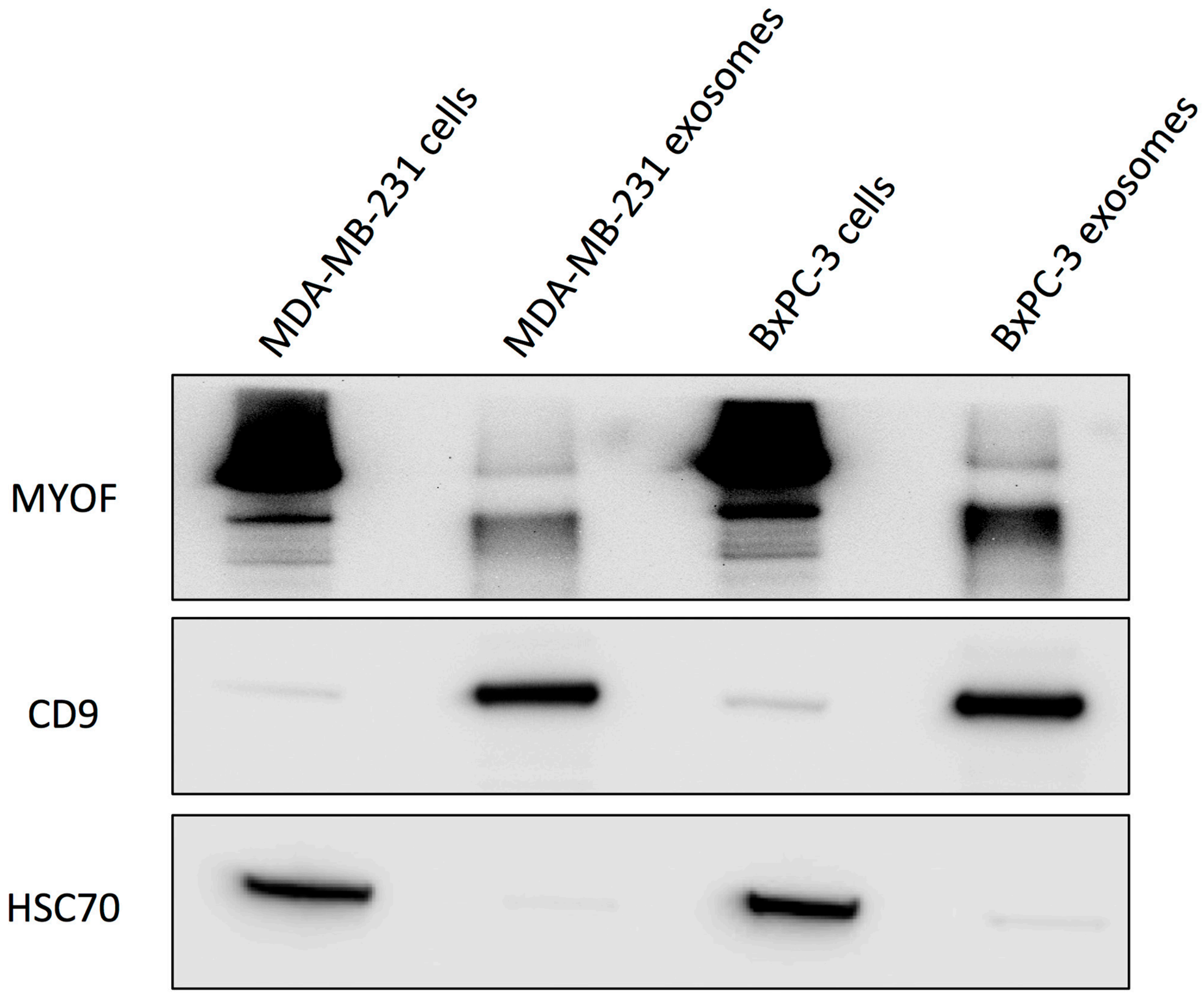

Supplementary Figure S2: Myoferlin expression in exosomes prepared by density-gradient ultracentrifugation.

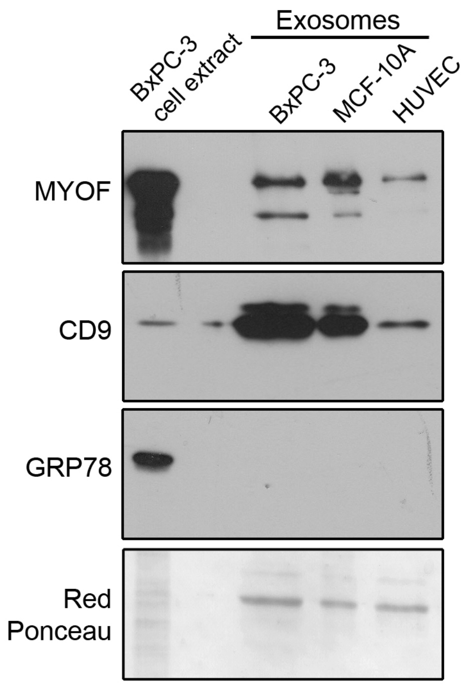

Supplementary Figure S3: Myoferlin is present in exosomes from non-cancerous cells. Myoferlin levels in exosome preparations from BxPC-3 (pancreatic cancer), MCF10A (non-transformed breast epithelial cells) and HUVEC (endothelial cells).

Supplementary Table S1: Proteomic analysis of exosomes isolated from myoferlin-silenced and control cancer cells.

See Supplementary File 1

Supplementary Table S2: Exosomal proteins modulated in absence of myoferlin.

See Supplementary File 2

Supplementary Table S3: Proteins modulated in common (MDA-MB-231 and BxPC-3 cells) following the myoferlin silencing.

See Supplementary File 3
