# Supplementary material for: Myoferlin is a novel exosomal protein and functional regulator of cancer-derived exosomes
Source: Oncotarget. 2016 Nov 10;7(50):83669–83. doi: 10.18632/oncotarget.13276 (PMC5347796; doi:10.18632/oncotarget.13276)
Supplement: Supplementary file 2 [file oncotarget-07-83669-s002.docx]

**Supplementary Table S1**

**Proteomic analysis of exosomes isolated from MDA-MB-231.**

| **Accession** | **Protein Description** | **Gene Name** | **CTRL/MYOF Ratio** |
| --- | --- | --- | --- |
| P62258 | 14-3-3 protein epsilon | YWHAE | 0.72 |
| P61981 | 14-3-3 protein gamma | YWHAG | CTRL |
| P31947 | 14-3-3 protein sigma | SFN | 0.62 |
| P63104 | 14-3-3 protein zeta/delta | YWHAZ | 0.85 |
| P09543 | 2,3-cyclic-nucleotide 3-phosphodiesterase | CNP | 1.10 |
| P62333 | 26S protease regulatory subunit 10B | PSMC6 | 3.01 |
| P62191 | 26S protease regulatory subunit 4 | PSMC1 | CTRL |
| P17980 | 26S protease regulatory subunit 6A | PSMC3 | CTRL |
| P43686 | 26S protease regulatory subunit 6B | PSMC4 | 4.51 |
| P35998 | 26S protease regulatory subunit 7 | PSMC2 | 2.49 |
| P62195 | 26S protease regulatory subunit 8 | PSMC5 | CTRL |
| Q99460 | 26S proteasome non-ATPase regulatory subunit 1 | PSMD1 | CTRL |
| O00231 | 26S proteasome non-ATPase regulatory subunit 11 | PSMD11 | 1.30 |
| Q13200 | 26S proteasome non-ATPase regulatory subunit 2 | PSMD2 | 1.78 |
| P48556 | 26S proteasome non-ATPase regulatory subunit 8 | PSMD8 | 3.68 |
| P62277 | 40S ribosomal protein S13 | RPS13 | CTRL |
| P62249 | 40S ribosomal protein S16 | RPS16 | CTRL |
| P15880 | 40S ribosomal protein S2 | RPS2 | CTRL |
| P61247 | 40S ribosomal protein S3a | RPS3A | CTRL |
| P62701 | 40S ribosomal protein S4, X isoform | RPS4X | CTRL |
| P62241 | 40S ribosomal protein S8 | RPS8 | CTRL |
| P46781 | 40S ribosomal protein S9 | RPS9 | 3.31 |
| P08865 | 40S ribosomal protein SA | RPSA | CTRL |
| P21589 | 5-nucleotidase | NT5E | 1.08 |
| P50914 | 60S ribosomal protein L14 | RPL14 | CTRL |
| P61313 | 60S ribosomal protein L15 | RPL15 | 2.65 |
| P18621 | 60S ribosomal protein L17 | RPL17 | CTRL |
| P83731 | 60S ribosomal protein L24 | RPL24 | CTRL |
| P36578 | 60S ribosomal protein L4 | RPL4 | 2.62 |
| P46777 | 60S ribosomal protein L5 | RPL5 | CTRL |
| Q02878 | 60S ribosomal protein L6 | RPL6 | 5.92 |
| P18124 | 60S ribosomal protein L7 | RPL7 | CTRL |
| P62424 | 60S ribosomal protein L7a | RPL7A | 5.21 |
| P11021 | 78 kDa glucose-regulated protein | HSPA5 | 2.56 |
| P61158 | Actin-related protein 3 | ACTR3 | CTRL |
| P07741 | Adenine phosphoribosyltransferase | APRT | 0.79 |
| P23526 | Adenosylhomocysteinase | AHCY | 0.87 |
| P84077 | ADP-ribosylation factor 1 | ARF1 | 1.78 |
| P18085 | ADP-ribosylation factor 4 | ARF4 | 1.71 |
| P62330 | ADP-ribosylation factor 6 | ARF6 | 1.91 |
| Q9NVJ2 | ADP-ribosylation factor-like protein 8B | ARL8B | 8.22 |
| O00468 | Agrin | AGRN | 2.83 |
| P14550 | Alcohol dehydrogenase [NADP(+)] | AKR1A1 | 0.65 |
| P00330 | Alcohol dehydrogenase 1 | ADH1 | 0.98 |
| O43707 | Alpha-actinin-4 | ACTN4 | 1.51 |
| P06733 | Alpha-enolase | ENO1 | 1.07 |
| P50995 | Annexin A11 | ANXA11 | 1.29 |
| P07355 | Annexin A2 | ANXA2 | 0.80 |
| P12429 | Annexin A3 | ANXA3 | CTRL |
| P08758 | Annexin A5 | ANXA5 | 1.24 |
| P08133 | Annexin A6 | ANXA6 | 1.17 |
| P20073 | Annexin A7 | ANXA7 | CTRL |
| P63010 | AP-2 complex subunit beta | AP2B1 | 2.64 |
| P04114 | Apolipoprotein B-100 | APOB | 0.59 |
| P00966 | Argininosuccinate synthase | ASS1 | 0.41 |
| Q8N5I2 | Arrestin domain-containing protein 1 | ARRDC1 | CTRL |
| P14868 | Aspartate--tRNA ligase, cytoplasmic | DARS | 1.33 |
| P53396 | ATP-citrate synthase | ACLY | 1.98 |
| Q01813 | ATP-dependent 6-phosphofructokinase, platelet type | PFKP | 2.60 |
| O00148 | ATP-dependent RNA helicase DDX39A | DDX39A | CTRL |
| O00571 | ATP-dependent RNA helicase DDX3X | DDX3X | CTRL |
| P98160 | Basement membrane-specific heparan sulfate proteoglycan core protein | HSPG2 | 1.50 |
| P35613 | Basigin | BSG | 0.72 |
| P61769 | Beta-2-microglobulin | B2M | 0.55 |
| P07814 | Bifunctional glutamate/proline--tRNA ligase | EPRS | 4.63 |
| Q9UQB8 | Brain-specific angiogenesis inhibitor 1-associated protein 2 | BAIAP2 | CTRL |
| Q5VW32 | BRO1 domain-containing protein BROX | BROX | CTRL |
| P11586 | C-1-tetrahydrofolate synthase, cytoplasmic | MTHFD1 | 1.29 |
| Q9Y376 | Calcium-binding protein 39 | CAB39 | 0.68 |
| P17655 | Calpain-2 catalytic subunit | CAPN2 | 0.70 |
| O15484 | Calpain-5 | CAPN5 | CTRL |
| Q8NEV1 | Casein kinase II subunit alpha 3 | CSNK2A3 | CTRL |
| P35222 | Catenin beta-1 | CTNNB1 | 3.62 |
| P07339 | Cathepsin D | CTSD | 0.83 |
| Q03135 | Caveolin-1 | CAV1 | 2.17 |
| P48509 | CD151 antigen | CD151 | 0.81 |
| Q9Y5K6 | CD2-associated protein | CD2AP | CTRL |
| P08962 | CD63 antigen | CD63 | CTRL |
| P21926 | CD9 antigen | CD9 | 1.22 |
| P60953 | Cell division control protein 42 homolog | CDC42 | 0.82 |
| Q16739 | Ceramide glucosyltransferase | UGCG | CTRL |
| O43633 | Charged multivesicular body protein 2a | CHMP2A | CTRL |
| Q8IWA5 | Choline transporter-like protein 2 | SLC44A2 | 3.68 |
| Q00610 | Clathrin heavy chain 1 | CLTC | 2.59 |
| P09497 | Clathrin light chain B | CLTB | CTRL |
| P00742 | Coagulation factor X | F10 | 1.02 |
| P20908 | Collagen alpha-1(V) chain | COL5A1 | 1.79 |
| P12109 | Collagen alpha-1(VI) chain | COL6A1 | 0.75 |
| P39060 | Collagen alpha-1(XVIII) chain | COL18A1 | 1.72 |
| Q99829 | Copine-1 | CPNE1 | CTRL |
| O75131 | Copine-3 | CPNE3 | 2.15 |
| Q86YQ8 | Copine-8 | CPNE8 | 3.29 |
| P31146 | Coronin-1A | CORO1A | 0.78 |
| Q9H5V8 | CUB domain-containing protein 1 | CDCP1 | 1.27 |
| P06493 | Cyclin-dependent kinase 1 | CDK1 | 3.00 |
| Q14204 | Cytoplasmic dynein 1 heavy chain 1 | DYNC1H1 | 3.08 |
| O00154 | Cytosolic acyl coenzyme A thioester hydrolase | ACOT7 | CTRL |
| Q14126 | Desmoglein-2 | DSG2 | 3.16 |
| P15924 | Desmoplakin | DSP | 0.54 |
| Q16555 | Dihydropyrimidinase-related protein 2 | DPYSL2 | 0.91 |
| Q9P265 | Disco-interacting protein 2 homolog B | DIP2B | 2.26 |
| O14672 | Disintegrin and metalloproteinase domain-containing protein 10 | ADAM10 | 1.27 |
| P11387 | DNA topoisomerase 1 | TOP1 | CTRL |
| P78527 | DNA-dependent protein kinase catalytic subunit | PRKDC | CTRL |
| O60884 | DnaJ homolog subfamily A member 2 | DNAJA2 | 2.82 |
| P25685 | DnaJ homolog subfamily B member 1 | DNAJB1 | 2.19 |
| P50570 | Dynamin-2 | DNM2 | 3.98 |
| Q14258 | E3 ubiquitin/ISG15 ligase TRIM25 | TRIM25 | CTRL |
| P22413 | Ectonucleotide pyrophosphatase/phosphodiesterase family member 1 | ENPP1 | 0.63 |
| O43854 | EGF-like repeat and discoidin I-like domain-containing protein 3 | EDIL3 | 1.54 |
| Q9NZN4 | EH domain-containing protein 2 | EHD2 | 2.20 |
| P26641 | Elongation factor 1-gamma | EEF1G | 2.55 |
| P13639 | Elongation factor 2 | EEF2 | 1.10 |
| P14625 | Endoplasmin | HSP90B1 | CTRL |
| P29317 | Ephrin type-A receptor 2 | EPHA2 | 1.28 |
| Q99808 | Equilibrative nucleoside transporter 1 | SLC29A1 | 1.20 |
| P60842 | Eukaryotic initiation factor 4A-I | EIF4A1 | 1.28 |
| P38919 | Eukaryotic initiation factor 4A-III | EIF4A3 | CTRL |
| P63241 | Eukaryotic translation initiation factor 5A-1 | EIF5A | 2.93 |
| P56537 | Eukaryotic translation initiation factor 6 | EIF6 | CTRL |
| P49327 | Fatty acid synthase | FASN | 1.78 |
| P02794 | Ferritin heavy chain | FTH1 | 2.63 |
| P02792 | Ferritin light chain | FTL | 1.97 |
| P02751 | Fibronectin | FN1 | 1.41 |
| P21333 | Filamin-A | FLNA | 1.31 |
| O75369 | Filamin-B | FLNB | 8.50 |
| O75955 | Flotillin-1 | FLOT1 | 4.22 |
| Q14254 | Flotillin-2 | FLOT2 | 4.25 |
| P04075 | Fructose-bisphosphate aldolase A | ALDOA | 0.76 |
| P09382 | Galectin-1 | LGALS1 | 0.74 |
| P11413 | Glucose-6-phosphate 1-dehydrogenase | G6PD | 5.14 |
| P06744 | Glucose-6-phosphate isomerase | GPI | 1.31 |
| Q06210 | Glutamine--fructose-6-phosphate aminotransferase [isomerizing] 1 | GFPT1 | 2.94 |
| Q7RTV2 | Glutathione S-transferase A5 | GSTA5 | 0.54 |
| P04406 | Glyceraldehyde-3-phosphate dehydrogenase | GAPDH | 1.32 |
| P62993 | Growth factor receptor-bound protein 2 | GRB2 | CTRL |
| P01111 | GTPase NRas | NRAS | 1.29 |
| P62826 | GTP-binding nuclear protein Ran | RAN | 0.76 |
| Q15382 | GTP-binding protein Rheb | RHEB | 2.32 |
| P04899 | Guanine nucleotide-binding protein G(i) subunit alpha-2 | GNAI2 | 0.88 |
| P62879 | Guanine nucleotide-binding protein G(I)/G(S)/G(T) subunit beta-2 | GNB2 | 1.32 |
| P63092 | Guanine nucleotide-binding protein G(s) subunit alpha isoforms short | GNAS | 1.38 |
| Q14344 | Guanine nucleotide-binding protein subunit alpha-13 | GNA13 | 1.16 |
| P63244 | Guanine nucleotide-binding protein subunit beta-2-like 1 | GNB2L1 | CTRL |
| P48723 | Heat shock 70 kDa protein 13 | HSPA13 | 0.74 |
| P08107 | Heat shock 70 kDa protein 1A/1B | HSPA1A | 1.59 |
| P11142 | Heat shock cognate 71 kDa protein | HSPA8 | 1.50 |
| Q92598 | Heat shock protein 105 kDa | HSPH1 | 2.69 |
| P04792 | Heat shock protein beta-1 | HSPB1 | 1.18 |
| P08238 | Heat shock protein HSP 90-beta | HSP90AB1 | 1.18 |
| P26927 | Hepatocyte growth factor-like protein | MST1 | MYOF |
| P61978 | Heterogeneous nuclear ribonucleoprotein K | HNRNPK | CTRL |
| P52272 | Heterogeneous nuclear ribonucleoprotein M | HNRNPM | CTRL |
| P30825 | High affinity cationic amino acid transporter 1 | SLC7A1 | 2.37 |
| P16403 | Histone H1.2 | HIST1H1C | 1.33 |
| Q16778 | Histone H2B type 2-E | HIST2H2BE | 1.43 |
| Q71DI3 | Histone H3.2 | HIST2H3A | 1.36 |
| P62805 | Histone H4 | HIST1H4A | 1.88 |
| P01892 | HLA class I histocompatibility antigen, A-2 alpha chain | HLA-A | 1.74 |
| Q04826 | HLA class I histocompatibility antigen, B-40 alpha chain | HLA-B | 0.71 |
| Q969P0 | Immunoglobulin superfamily member 8 | IGSF8 | CTRL |
| P12268 | Inosine-5-monophosphate dehydrogenase 2 | IMPDH2 | 3.36 |
| P17301 | Integrin alpha-2 | ITGA2 | 0.89 |
| P08648 | Integrin alpha-5 | ITGA5 | 0.98 |
| P23229 | Integrin alpha-6 | ITGA6 | 0.84 |
| P06756 | Integrin alpha-V | ITGAV | 1.52 |
| P05106 | Integrin beta-3 | ITGB3 | 1.68 |
| P16144 | Integrin beta-4 | ITGB4 | 0.86 |
| Q13418 | Integrin-linked protein kinase | ILK | 1.09 |
| O75874 | Isocitrate dehydrogenase [NADP] cytoplasmic | IDH1 | CTRL |
| P41252 | Isoleucine--tRNA ligase, cytoplasmic | IARS | CTRL |
| P53990 | IST1 homolog | IST1 | 1.52 |
| P14923 | Junction plakoglobin | JUP | 0.67 |
| Q08431 | Lactadherin | MFGE8 | 2.12 |
| Q9P2J5 | Leucine--tRNA ligase, cytoplasmic | LARS | CTRL |
| Q86X29 | Lipolysis-stimulated lipoprotein receptor | LSR | 3.77 |
| P00338 | L-lactate dehydrogenase A chain | LDHA | 0.85 |
| P07195 | L-lactate dehydrogenase B chain | LDHB | 0.81 |
| O60488 | Long-chain-fatty-acid--CoA ligase 4 | ACSL4 | 2.14 |
| Q7Z4F1 | Low-density lipoprotein receptor-related protein 10 | LRP10 | CTRL |
| Q9H3U5 | Major facilitator superfamily domain-containing protein 1 | MFSD1 | CTRL |
| Q14764 | Major vault protein | MVP | 2.18 |
| P20774 | Mimecan | OGN | 0.58 |
| O15427 | Monocarboxylate transporter 4 | SLC16A3 | 1.14 |
| O95297 | Myelin protein zero-like protein 1 | MPZL1 | 0.27 |
| Q96S97 | Myeloid-associated differentiation marker | MYADM | CTRL |
| Q9NZM1 | Myoferlin | MYOF | CTRL |
| Q9Y2A7 | Nck-associated protein 1 | NCKAP1 | CTRL |
| Q09666 | Neuroblast differentiation-associated protein AHNAK | AHNAK | 1.20 |
| O14786 | Neuropilin-1 | NRP1 | 1.37 |
| Q15758 | Neutral amino acid transporter B(0) | SLC1A5 | 1.84 |
| Q96TA1 | Niban-like protein 1 | FAM129B | MYOF |
| P43490 | Nicotinamide phosphoribosyltransferase | NAMPT | 2.53 |
| O15118 | Niemann-Pick C1 protein | NPC1 | 3.85 |
| P22392 | Nucleoside diphosphate kinase B | NME2 | 0.91 |
| Q99650 | Oncostatin-M-specific receptor subunit beta | OSMR | CTRL |
| Q9C0B5 | Palmitoyltransferase ZDHHC5 | ZDHHC5 | CTRL |
| P62937 | Peptidyl-prolyl cis-trans isomerase A | PPIA | 1.17 |
| Q06830 | Peroxiredoxin-1 | PRDX1 | 1.16 |
| Q9BTU6 | Phosphatidylinositol 4-kinase type 2-alpha | PI4K2A | CTRL |
| P20020 | Plasma membrane calcium-transporting ATPase 1 | ATP2B1 | 1.36 |
| P05121 | Plasminogen activator inhibitor 1 | SERPINE1 | CTRL |
| Q15149 | Plectin | PLEC | CTRL |
| O15031 | Plexin-B2 | PLXNB2 | 1.48 |
| Q9UKK3 | Poly [ADP-ribose] polymerase 4 | PARP4 | 4.87 |
| P11940 | Polyadenylate-binding protein 1 | PABPC1 | CTRL |
| P0CG47 | Polyubiquitin-B | UBB | 1.62 |
| Q8WUM4 | Programmed cell death 6-interacting protein | PDCD6IP | 1.33 |
| O75340 | Programmed cell death protein 6 | PDCD6 | 2.18 |
| O00622 | Protein CYR61 | CYR61 | 3.88 |
| P07237 | Protein disulfide-isomerase | P4HB | CTRL |
| Q92734 | Protein TFG | TFG | CTRL |
| Q9C0H2 | Protein tweety homolog 3 | TTYH3 | 2.15 |
| P12931 | Proto-oncogene tyrosine-protein kinase Src | SRC | 2.35 |
| Q9Y315 | Putative deoxyribose-phosphate aldolase | DERA | CTRL |
| Q5VTE0 | Putative elongation factor 1-alpha-like 3 | EEF1A1P5 | 1.30 |
| O43143 | Putative pre-mRNA-splicing factor ATP-dependent RNA helicase DHX15 | DHX15 | CTRL |
| P14618 | Pyruvate kinase PKM | PKM | 0.87 |
| P50395 | Rab GDP dissociation inhibitor beta | GDI2 | 1.83 |
| Q14699 | Raftlin | RFTN1 | 2.35 |
| P46940 | Ras GTPase-activating-like protein IQGAP1 | IQGAP1 | 3.04 |
| Q15404 | Ras suppressor protein 1 | RSU1 | 0.50 |
| P63000 | Ras-related C3 botulinum toxin substrate 1 | RAC1 | 0.78 |
| P15153 | Ras-related C3 botulinum toxin substrate 2 | RAC2 | 3.12 |
| P61026 | Ras-related protein Rab-10 | RAB10 | 1.23 |
| Q15907 | Ras-related protein Rab-11B | RAB11B | 1.13 |
| P61106 | Ras-related protein Rab-14 | RAB14 | 1.29 |
| Q9H0U4 | Ras-related protein Rab-1B | RAB1B | 0.87 |
| Q9UL26 | Ras-related protein Rab-22A | RAB22A | 4.12 |
| P61019 | Ras-related protein Rab-2A | RAB2A | CTRL |
| P61020 | Ras-related protein Rab-5B | RAB5B | 2.33 |
| P51148 | Ras-related protein Rab-5C | RAB5C | 1.74 |
| P51149 | Ras-related protein Rab-7a | RAB7A | 1.55 |
| P61006 | Ras-related protein Rab-8A | RAB8A | 0.78 |
| P61224 | Ras-related protein Rap-1b | RAP1B | 0.83 |
| P10301 | Ras-related protein R-Ras | RRAS | 1.92 |
| P62070 | Ras-related protein R-Ras2 | RRAS2 | 2.29 |
| P18433 | Receptor-type tyrosine-protein phosphatase alpha | PTPRA | CTRL |
| P10586 | Receptor-type tyrosine-protein phosphatase F | PTPRF | 3.97 |
| P23470 | Receptor-type tyrosine-protein phosphatase gamma | PTPRG | CTRL |
| Q15262 | Receptor-type tyrosine-protein phosphatase kappa | PTPRK | 4.22 |
| Q8NFJ5 | Retinoic acid-induced protein 3 | GPRC5A | 4.24 |
| Q9Y265 | RuvB-like 1 | RUVBL1 | 4.07 |
| Q9Y230 | RuvB-like 2 | RUVBL2 | CTRL |
| O14828 | Secretory carrier-associated membrane protein 3 | SCAMP3 | 3.23 |
| Q13501 | Sequestosome-1 | SQSTM1 | CTRL |
| O95747 | Serine/threonine-protein kinase OSR1 | OXSR1 | 4.35 |
| P30153 | Serine/threonine-protein phosphatase 2A 65 kDa regulatory subunit A alpha isoform | PPP2R1A | 0.74 |
| Q92783 | Signal transducing adapter molecule 1 | STAM | 3.98 |
| P62318 | Small nuclear ribonucleoprotein Sm D3 | SNRPD3 | CTRL |
| Q8NCG7 | Sn1-specific diacylglycerol lipase beta | DAGLB | CTRL |
| P05023 | Sodium/potassium-transporting ATPase subunit alpha-1 | ATP1A1 | 1.27 |
| P05026 | Sodium/potassium-transporting ATPase subunit beta-1 | ATP1B1 | 1.30 |
| P54709 | Sodium/potassium-transporting ATPase subunit beta-3 | ATP1B3 | CTRL |
| Q9H2H9 | Sodium-coupled neutral amino acid transporter 1 | SLC38A1 | 1.50 |
| P55011 | Solute carrier family 12 member 2 | SLC12A2 | 2.76 |
| P11166 | Solute carrier family 2, facilitated glucose transporter member 1 | SLC2A1 | 1.71 |
| Q8NBI5 | Solute carrier family 43 member 3 | SLC43A3 | 1.24 |
| Q15036 | Sorting nexin-17 | SNX17 | CTRL |
| O60493 | Sorting nexin-3 | SNX3 | 3.65 |
| P31948 | Stress-induced-phosphoprotein 1 | STIP1 | 1.09 |
| Q6UWP8 | Suprabasin | SBSN | 0.65 |
| O43752 | Syntaxin-6 | STX6 | CTRL |
| O00560 | Syntenin-1 | SDCBP | 1.80 |
| Q9Y490 | Talin-1 | TLN1 | 1.12 |
| Q86VP1 | Tax1-binding protein 1 | TAX1BP1 | CTRL |
| P78371 | T-complex protein 1 subunit beta | CCT2 | 1.26 |
| P50991 | T-complex protein 1 subunit delta | CCT4 | 1.46 |
| P48643 | T-complex protein 1 subunit epsilon | CCT5 | 2.08 |
| Q99832 | T-complex protein 1 subunit eta | CCT7 | CTRL |
| P49368 | T-complex protein 1 subunit gamma | CCT3 | 1.17 |
| Q9P273 | Teneurin-3 | TENM3 | CTRL |
| Q8NG11 | Tetraspanin-14 | TSPAN14 | 4.47 |
| P37173 | TGF-beta receptor type-2 | TGFBR2 | CTRL |
| P10599 | Thioredoxin | TXN | CTRL |
| Q9H3M7 | Thioredoxin-interacting protein | TXNIP | CTRL |
| P13726 | Tissue factor | F3 | CTRL |
| P02786 | Transferrin receptor protein 1 | TFRC | 3.02 |
| Q15582 | Transforming growth factor-beta-induced protein ig-h3 | TGFBI | 0.36 |
| P37802 | Transgelin-2 | TAGLN2 | 0.92 |
| P55072 | Transitional endoplasmic reticulum ATPase | VCP | 1.43 |
| Q92616 | Translational activator GCN1 | GCN1L1 | CTRL |
| P13693 | Translationally-controlled tumor protein | TPT1 | 0.87 |
| P60174 | Triosephosphate isomerase | TPI1 | 1.03 |
| P29144 | Tripeptidyl-peptidase 2 | TPP2 | 2.77 |
| Q9BQE3 | Tubulin alpha-1C chain | TUBA1C | 1.99 |
| P68366 | Tubulin alpha-4A chain | TUBA4A | 1.31 |
| P07437 | Tubulin beta chain | TUBB | 1.82 |
| P68371 | Tubulin beta-4B chain | TUBB4B | 1.59 |
| Q99816 | Tumor susceptibility gene 101 protein | TSG101 | 5.07 |
| P23458 | Tyrosine-protein kinase JAK1 | JAK1 | CTRL |
| P30530 | Tyrosine-protein kinase receptor UFO | AXL | 2.61 |
| P07947 | Tyrosine-protein kinase Yes | YES1 | CTRL |
| P09012 | U1 small nuclear ribonucleoprotein A | SNRPA | MYOF |
| A0AVT1 | Ubiquitin-like modifier-activating enzyme 6 | UBA6 | CTRL |
| O60701 | UDP-glucose 6-dehydrogenase | UGDH | CTRL |
| O00159 | Unconventional myosin-Ic | MYO1C | 2.69 |
| P00749 | Urokinase-type plasminogen activator | PLAU | 0.66 |
| Q16851 | UTP--glucose-1-phosphate uridylyltransferase | UGP2 | 0.68 |
| Q9UK41 | Vacuolar protein sorting-associated protein 28 homolog | VPS28 | 6.45 |
| Q9H9H4 | Vacuolar protein sorting-associated protein 37B | VPS37B | CTRL |
| O75351 | Vacuolar protein sorting-associated protein 4B | VPS4B | 6.04 |
| P51809 | Vesicle-associated membrane protein 7 | VAMP7 | CTRL |
| P08670 | Vimentin | VIM | 0.91 |
| P18206 | Vinculin | VCL | 0.74 |
| P13010 | X-ray repair cross-complementing protein 5 | XRCC5 | CTRL |
| P12956 | X-ray repair cross-complementing protein 6 | XRCC6 | 2.50 |

**Proteomic analysis of exosomes isolated from BxPC3.**

| **Accession** | **Protein Description** | **Gene Name** | **CTRL/MYOF Ratio** |
| --- | --- | --- | --- |
| P31946 | 14-3-3 protein beta/alpha | YWHAB | 0.95 |
| P62258 | 14-3-3 protein epsilon | YWHAE | 1.04 |
| P61981 | 14-3-3 protein gamma | YWHAG | 0.98 |
| P31947 | 14-3-3 protein sigma | SFN | 0.89 |
| P63104 | 14-3-3 protein zeta/delta | YWHAZ | 1.20 |
| P09543 | 2,3-cyclic-nucleotide 3-phosphodiesterase | CNP | 0.69 |
| P62191 | 26S protease regulatory subunit 4 | PSMC1 | CTRL |
| P43686 | 26S protease regulatory subunit 6B | PSMC4 | 0.43 |
| O00231 | 26S proteasome non-ATPase regulatory subunit 11 | PSMD11 | 0.82 |
| O00232 | 26S proteasome non-ATPase regulatory subunit 12 | PSMD12 | 0.37 |
| Q9UNM6 | 26S proteasome non-ATPase regulatory subunit 13 | PSMD13 | 0.49 |
| Q13200 | 26S proteasome non-ATPase regulatory subunit 2 | PSMD2 | 0.90 |
| P48556 | 26S proteasome non-ATPase regulatory subunit 8 | PSMD8 | 0.54 |
| P25398 | 40S ribosomal protein S12 | RPS12 | MYOF |
| P62277 | 40S ribosomal protein S13 | RPS13 | MYOF |
| P62249 | 40S ribosomal protein S16 | RPS16 | 0.51 |
| P15880 | 40S ribosomal protein S2 | RPS2 | 0.75 |
| P60866 | 40S ribosomal protein S20 | RPS20 | 0.23 |
| P23396 | 40S ribosomal protein S3 | RPS3 | 0.23 |
| P61247 | 40S ribosomal protein S3a | RPS3A | 0.38 |
| P62701 | 40S ribosomal protein S4, X isoform | RPS4X | 0.40 |
| P46781 | 40S ribosomal protein S9 | RPS9 | 0.99 |
| P08865 | 40S ribosomal protein SA | RPSA | 0.32 |
| P08195 | 4F2 cell-surface antigen heavy chain | SLC3A2 | 1.25 |
| P21589 | 5-nucleotidase | NT5E | 0.95 |
| P05388 | 60S acidic ribosomal protein P0 | RPLP0 | MYOF |
| P27635 | 60S ribosomal protein L10 | RPL10 | 0.41 |
| P62906 | 60S ribosomal protein L10a | RPL10A | MYOF |
| P50914 | 60S ribosomal protein L14 | RPL14 | 0.78 |
| P18621 | 60S ribosomal protein L17 | RPL17 | 0.52 |
| P39023 | 60S ribosomal protein L3 | RPL3 | 0.95 |
| P36578 | 60S ribosomal protein L4 | RPL4 | 1.04 |
| Q02878 | 60S ribosomal protein L6 | RPL6 | 0.35 |
| P18124 | 60S ribosomal protein L7 | RPL7 | 0.99 |
| P52209 | 6-phosphogluconate dehydrogenase, decarboxylating | PGD | 0.57 |
| P11021 | 78 kDa glucose-regulated protein | HSPA5 | 3.66 |
| P63261 | Actin, cytoplasmic 2 | ACTG1 | 0.71 |
| P61160 | Actin-related protein 2 | ACTR2 | 0.52 |
| O15144 | Actin-related protein 2/3 complex subunit 2 | ARPC2 | 0.42 |
| O15145 | Actin-related protein 2/3 complex subunit 3 | ARPC3 | 0.49 |
| P61158 | Actin-related protein 3 | ACTR3 | 0.37 |
| P55263 | Adenosine kinase | ADK | 0.42 |
| P23526 | Adenosylhomocysteinase | AHCY | 1.02 |
| P84077 | ADP-ribosylation factor 1 | ARF1 | 0.89 |
| P18085 | ADP-ribosylation factor 4 | ARF4 | 1.28 |
| P62330 | ADP-ribosylation factor 6 | ARF6 | 0.77 |
| Q9NVJ2 | ADP-ribosylation factor-like protein 8B | ARL8B | 1.85 |
| O00468 | Agrin | AGRN | 1.48 |
| P14550 | Alcohol dehydrogenase [NADP(+)] | AKR1A1 | 1.25 |
| P11766 | Alcohol dehydrogenase class-3 | ADH5 | MYOF |
| P47895 | Aldehyde dehydrogenase family 1 member A3 | ALDH1A3 | 0.61 |
| P30838 | Aldehyde dehydrogenase, dimeric NADP-preferring | ALDH3A1 | 1.31 |
| Q04828 | Aldo-keto reductase family 1 member C1 | AKR1C1 | 0.38 |
| P12814 | Alpha-actinin-1 | ACTN1 | 1.32 |
| O43707 | Alpha-actinin-4 | ACTN4 | 1.98 |
| P06733 | Alpha-enolase | ENO1 | 1.28 |
| P04083 | Annexin A1 | ANXA1 | 1.19 |
| P50995 | Annexin A11 | ANXA11 | 0.63 |
| P07355 | Annexin A2 | ANXA2 | 1.20 |
| P12429 | Annexin A3 | ANXA3 | 1.12 |
| P09525 | Annexin A4 | ANXA4 | 1.08 |
| P08758 | Annexin A5 | ANXA5 | 1.14 |
| P20073 | Annexin A7 | ANXA7 | 0.64 |
| Q5XXA6 | Anoctamin-1 | ANO1 | 1.44 |
| O95782 | AP-2 complex subunit alpha-1 | AP2A1 | 1.26 |
| P63010 | AP-2 complex subunit beta | AP2B1 | 1.12 |
| Q96CW1 | AP-2 complex subunit mu | AP2M1 | 0.93 |
| P04424 | Argininosuccinate lyase | ASL | MYOF |
| P00966 | Argininosuccinate synthase | ASS1 | 0.75 |
| Q8N5I2 | Arrestin domain-containing protein 1 | ARRDC1 | 1.26 |
| P08243 | Asparagine synthetase [glutamine-hydrolyzing] | ASNS | CTRL |
| P14868 | Aspartate--tRNA ligase, cytoplasmic | DARS | 0.36 |
| P06576 | ATP synthase subunit beta, mitochondrial | ATP5B | MYOF |
| P61221 | ATP-binding cassette sub-family E member 1 | ABCE1 | MYOF |
| P53396 | ATP-citrate synthase | ACLY | 0.76 |
| Q01813 | ATP-dependent 6-phosphofructokinase, platelet type | PFKP | 0.30 |
| Q08211 | ATP-dependent RNA helicase A | DHX9 | 0.22 |
| O00148 | ATP-dependent RNA helicase DDX39A | DDX39A | 0.33 |
| O00571 | ATP-dependent RNA helicase DDX3X | DDX3X | 0.40 |
| Q9H4G0 | Band 4.1-like protein 1 | EPB41L1 | 1.54 |
| P98160 | Basement membrane-specific heparan sulfate proteoglycan core protein | HSPG2 | 1.13 |
| P35613 | Basigin | BSG | 1.14 |
| P61769 | Beta-2-microglobulin | B2M | 0.20 |
| P07686 | Beta-hexosaminidase subunit beta | HEXB | 1.80 |
| P07814 | Bifunctional glutamate/proline--tRNA ligase | EPRS | MYOF |
| P31939 | Bifunctional purine biosynthesis protein PURH | ATIC | 0.47 |
| Q9UQB8 | Brain-specific angiogenesis inhibitor 1-associated protein 2 | BAIAP2 | 0.78 |
| Q5VW32 | BRO1 domain-containing protein BROX | BROX | 0.81 |
| P11586 | C-1-tetrahydrofolate synthase, cytoplasmic | MTHFD1 | 0.45 |
| P12830 | Cadherin-1 | CDH1 | 0.75 |
| P22223 | Cadherin-3 | CDH3 | 0.76 |
| Q99653 | Calcineurin B homologous protein 1 | CHP1 | 1.19 |
| Q9Y376 | Calcium-binding protein 39 | CAB39 | 1.07 |
| O15484 | Calpain-5 | CAPN5 | 1.36 |
| P10644 | cAMP-dependent protein kinase type I-alpha regulatory subunit | PRKAR1A | CTRL |
| Q9HCP0 | Casein kinase I isoform gamma-1 | CSNK1G1 | 0.43 |
| Q9Y6M4 | Casein kinase I isoform gamma-3 | CSNK1G3 | MYOF |
| Q8NEV1 | Casein kinase II subunit alpha 3 | CSNK2A3 | 1.25 |
| P35221 | Catenin alpha-1 | CTNNA1 | 0.81 |
| P35222 | Catenin beta-1 | CTNNB1 | 0.77 |
| O60716 | Catenin delta-1 | CTNND1 | 0.72 |
| P07339 | Cathepsin D | CTSD | 1.24 |
| P11717 | Cation-independent mannose-6-phosphate receptor | IGF2R | 1.20 |
| Q03135 | Caveolin-1 | CAV1 | 1.77 |
| Q6YHK3 | CD109 antigen | CD109 | 1.07 |
| P48509 | CD151 antigen | CD151 | 0.68 |
| Q13740 | CD166 antigen | ALCAM | 0.81 |
| Q9Y5K6 | CD2-associated protein | CD2AP | 0.98 |
| P16070 | CD44 antigen | CD44 | 1.29 |
| P08962 | CD63 antigen | CD63 | 2.29 |
| P27701 | CD82 antigen | CD82 | 0.95 |
| P21926 | CD9 antigen | CD9 | 1.03 |
| P60953 | Cell division control protein 42 homolog | CDC42 | 1.06 |
| Q53EZ4 | Centrosomal protein of 55 kDa | CEP55 | 1.28 |
| Q9HD42 | Charged multivesicular body protein 1a | CHMP1A | 0.51 |
| O43633 | Charged multivesicular body protein 2a | CHMP2A | 0.41 |
| Q9BY43 | Charged multivesicular body protein 4a | CHMP4A | 0.52 |
| Q9H444 | Charged multivesicular body protein 4b | CHMP4B | 0.48 |
| O00299 | Chloride intracellular channel protein 1 | CLIC1 | 0.77 |
| Q9Y696 | Chloride intracellular channel protein 4 | CLIC4 | 2.11 |
| Q8WWI5 | Choline transporter-like protein 1 | SLC44A1 | 0.68 |
| Q8IWA5 | Choline transporter-like protein 2 | SLC44A2 | 1.42 |
| O75390 | Citrate synthase, mitochondrial | CS | MYOF |
| Q00610 | Clathrin heavy chain 1 | CLTC | 1.13 |
| P09496 | Clathrin light chain A | CLTA | 1.30 |
| O95832 | Claudin-1 | CLDN1 | 0.50 |
| O14493 | Claudin-4 | CLDN4 | 0.70 |
| P53618 | Coatomer subunit beta | COPB1 | 0.36 |
| P23528 | Cofilin-1 | CFL1 | 0.65 |
| P12109 | Collagen alpha-1(VI) chain | COL6A1 | 0.97 |
| Q99715 | Collagen alpha-1(XII) chain | COL12A1 | 3.49 |
| Q9UMD9 | Collagen alpha-1(XVII) chain | COL17A1 | 0.53 |
| P08174 | Complement decay-accelerating factor | CD55 | MYOF |
| Q12860 | Contactin-1 | CNTN1 | 2.16 |
| Q99829 | Copine-1 | CPNE1 | 0.71 |
| Q86YQ8 | Copine-8 | CPNE8 | 0.58 |
| Q9H5V8 | CUB domain-containing protein 1 | CDCP1 | 0.78 |
| P06493 | Cyclin-dependent kinase 1 | CDK1 | 0.64 |
| Q9H1C7 | Cysteine-rich and transmembrane domain-containing protein 1 | CYSTM1 | CTRL |
| Q9UPY5 | Cystine/glutamate transporter | SLC7A11 | 1.16 |
| Q14204 | Cytoplasmic dynein 1 heavy chain 1 | DYNC1H1 | 0.45 |
| Q7L576 | Cytoplasmic FMR1-interacting protein 1 | CYFIP1 | 0.71 |
| O00154 | Cytosolic acyl coenzyme A thioester hydrolase | ACOT7 | MYOF |
| O43175 | D-3-phosphoglycerate dehydrogenase | PHGDH | 0.85 |
| Q14126 | Desmoglein-2 | DSG2 | 0.86 |
| P32926 | Desmoglein-3 | DSG3 | 0.90 |
| P15924 | Desmoplakin | DSP | 0.80 |
| Q9P265 | Disco-interacting protein 2 homolog B | DIP2B | 0.77 |
| O14672 | Disintegrin and metalloproteinase domain-containing protein 10 | ADAM10 | 0.87 |
| Q13443 | Disintegrin and metalloproteinase domain-containing protein 9 | ADAM9 | 0.45 |
| P31689 | DnaJ homolog subfamily A member 1 | DNAJA1 | 0.33 |
| Q9H3Z4 | DnaJ homolog subfamily C member 5 | DNAJC5 | 1.36 |
| Q02750 | Dual specificity mitogen-activated protein kinase kinase 1 | MAP2K1 | 0.29 |
| Q96J02 | E3 ubiquitin-protein ligase Itchy homolog | ITCH | 0.99 |
| O95834 | Echinoderm microtubule-associated protein-like 2 | EML2 | CTRL |
| Q9H4M9 | EH domain-containing protein 1 | EHD1 | 0.75 |
| Q9NZN4 | EH domain-containing protein 2 | EHD2 | 0.99 |
| Q9H223 | EH domain-containing protein 4 | EHD4 | 0.60 |
| P26641 | Elongation factor 1-gamma | EEF1G | 0.75 |
| P13639 | Elongation factor 2 | EEF2 | 0.48 |
| P00924 | Enolase 1 | ENO1 | 0.82 |
| P29317 | Ephrin type-A receptor 2 | EPHA2 | 1.22 |
| P54760 | Ephrin type-B receptor 4 | EPHB4 | 0.98 |
| P00533 | Epidermal growth factor receptor | EGFR | 0.84 |
| P16422 | Epithelial cell adhesion molecule | EPCAM | 1.07 |
| Q08345 | Epithelial discoidin domain-containing receptor 1 | DDR1 | 1.01 |
| Q99808 | Equilibrative nucleoside transporter 1 | SLC29A1 | 1.02 |
| P27105 | Erythrocyte band 7 integral membrane protein | STOM | 0.31 |
| P60842 | Eukaryotic initiation factor 4A-I | EIF4A1 | 0.57 |
| P38919 | Eukaryotic initiation factor 4A-III | EIF4A3 | MYOF |
| Q14152 | Eukaryotic translation initiation factor 3 subunit A | EIF3A | MYOF |
| P63241 | Eukaryotic translation initiation factor 5A-1 | EIF5A | 0.69 |
| P56537 | Eukaryotic translation initiation factor 6 | EIF6 | MYOF |
| P55060 | Exportin-2 | CSE1L | 1.04 |
| P52907 | F-actin-capping protein subunit alpha-1 | CAPZA1 | MYOF |
| Q16658 | Fascin | FSCN1 | 0.55 |
| P49327 | Fatty acid synthase | FASN | 0.55 |
| Q96NE9 | FERM domain-containing protein 6 | FRMD6 | 0.43 |
| Q9BQL6 | Fermitin family homolog 1 | FERMT1 | 0.78 |
| Q86UX7 | Fermitin family homolog 3 | FERMT3 | 0.49 |
| P02794 | Ferritin heavy chain | FTH1 | 1.75 |
| P02792 | Ferritin light chain | FTL | 1.37 |
| Q14512 | Fibroblast growth factor-binding protein 1 | FGFBP1 | 1.53 |
| P02751 | Fibronectin | FN1 | 2.25 |
| P21333 | Filamin-A | FLNA | 0.75 |
| O75369 | Filamin-B | FLNB | 0.19 |
| O75955 | Flotillin-1 | FLOT1 | 1.89 |
| Q14254 | Flotillin-2 | FLOT2 | 2.09 |
| P04075 | Fructose-bisphosphate aldolase A | ALDOA | 0.99 |
| P09382 | Galectin-1 | LGALS1 | 0.17 |
| P17931 | Galectin-3 | LGALS3 | 1.38 |
| Q08380 | Galectin-3-binding protein | LGALS3BP | 0.79 |
| P29033 | Gap junction beta-2 protein | GJB2 | 0.69 |
| P11413 | Glucose-6-phosphate 1-dehydrogenase | G6PD | 1.04 |
| P06744 | Glucose-6-phosphate isomerase | GPI | 2.12 |
| Q7RTV2 | Glutathione S-transferase A5 | GSTA5 | 0.44 |
| P09211 | Glutathione S-transferase P | GSTP1 | 1.32 |
| P04406 | Glyceraldehyde-3-phosphate dehydrogenase | GAPDH | 1.01 |
| P41250 | Glycine--tRNA ligase | GARS | MYOF |
| P11216 | Glycogen phosphorylase, brain form | PYGB | MYOF |
| P06737 | Glycogen phosphorylase, liver form | PYGL | 1.16 |
| P35052 | Glypican-1 | GPC1 | 1.64 |
| P49915 | GMP synthase [glutamine-hydrolyzing] | GMPS | MYOF |
| Q7Z5G4 | Golgin subfamily A member 7 | GOLGA7 | 0.49 |
| Q9Y653 | G-protein coupled receptor 56 | GPR56 | 1.03 |
| Q9NQ84 | G-protein coupled receptor family C group 5 member C | GPRC5C | 0.40 |
| P62993 | Growth factor receptor-bound protein 2 | GRB2 | 1.09 |
| P01111 | GTPase NRas | NRAS | 0.93 |
| P62826 | GTP-binding nuclear protein Ran | RAN | 0.64 |
| P04899 | Guanine nucleotide-binding protein G(i) subunit alpha-2 | GNAI2 | 0.90 |
| P62879 | Guanine nucleotide-binding protein G(I)/G(S)/G(T) subunit beta-2 | GNB2 | 0.95 |
| P63092 | Guanine nucleotide-binding protein G(s) subunit alpha isoforms short | GNAS | 1.01 |
| P63244 | Guanine nucleotide-binding protein subunit beta-2-like 1 | GNB2L1 | 0.30 |
| P08107 | Heat shock 70 kDa protein 1A/1B | HSPA1A | 0.88 |
| P11142 | Heat shock cognate 71 kDa protein | HSPA8 | 0.84 |
| Q92598 | Heat shock protein 105 kDa | HSPH1 | 1.25 |
| P04792 | Heat shock protein beta-1 | HSPB1 | 0.84 |
| P08238 | Heat shock protein HSP 90-beta | HSP90AB1 | 0.80 |
| O14792 | Heparan sulfate glucosamine 3-O-sulfotransferase 1 | HS3ST1 | 1.30 |
| P08581 | Hepatocyte growth factor receptor | MET | 1.18 |
| P09651 | Heterogeneous nuclear ribonucleoprotein A1 | HNRNPA1 | 0.47 |
| Q14103 | Heterogeneous nuclear ribonucleoprotein D0 | HNRNPD | 2.20 |
| P61978 | Heterogeneous nuclear ribonucleoprotein K | HNRNPK | 0.32 |
| P22626 | Heterogeneous nuclear ribonucleoproteins A2/B1 | HNRNPA2B1 | 1.00 |
| P30825 | High affinity cationic amino acid transporter 1 | SLC7A1 | 1.38 |
| Q16778 | Histone H2B type 2-E | HIST2H2BE | 2.48 |
| Q71DI3 | Histone H3.2 | HIST2H3A | 1.45 |
| P62805 | Histone H4 | HIST1H4A | 2.02 |
| P30455 | HLA class I histocompatibility antigen, A-36 alpha chain | HLA-A | 0.90 |
| O75054 | Immunoglobulin superfamily member 3 | IGSF3 | 0.85 |
| Q969P0 | Immunoglobulin superfamily member 8 | IGSF8 | 1.06 |
| P52292 | Importin subunit alpha-1 | KPNA2 | MYOF |
| Q14974 | Importin subunit beta-1 | KPNB1 | 0.42 |
| Q13308 | Inactive tyrosine-protein kinase 7 | PTK7 | 2.17 |
| P17301 | Integrin alpha-2 | ITGA2 | 1.07 |
| P26006 | Integrin alpha-3 | ITGA3 | 0.95 |
| P08648 | Integrin alpha-5 | ITGA5 | 0.34 |
| P23229 | Integrin alpha-6 | ITGA6 | 0.91 |
| P06756 | Integrin alpha-V | ITGAV | 0.74 |
| P05556 | Integrin beta-1 | ITGB1 | 0.97 |
| P05106 | Integrin beta-3 | ITGB3 | 0.79 |
| P16144 | Integrin beta-4 | ITGB4 | 0.87 |
| P18564 | Integrin beta-6 | ITGB6 | 1.05 |
| Q13418 | Integrin-linked protein kinase | ILK | 1.11 |
| Q9NPH3 | Interleukin-1 receptor accessory protein | IL1RAP | MYOF |
| O15554 | Intermediate conductance calcium-activated potassium channel protein 4 | KCNN4 | CTRL |
| O75874 | Isocitrate dehydrogenase [NADP] cytoplasmic | IDH1 | 2.76 |
| P41252 | Isoleucine--tRNA ligase, cytoplasmic | IARS | 0.29 |
| P53990 | IST1 homolog | IST1 | 0.73 |
| P14923 | Junction plakoglobin | JUP | 0.86 |
| Q9Y624 | Junctional adhesion molecule A | F11R | 0.78 |
| Q96J84 | Kin of IRRE-like protein 1 | KIRREL | 3.15 |
| Q02241 | Kinesin-like protein KIF23 | KIF23 | 0.94 |
| O43278 | Kunitz-type protease inhibitor 1 | SPINT1 | 0.34 |
| Q08431 | Lactadherin | MFGE8 | 1.04 |
| P07942 | Laminin subunit beta-1 | LAMB1 | 1.31 |
| Q13751 | Laminin subunit beta-3 | LAMB3 | 0.21 |
| P11047 | Laminin subunit gamma-1 | LAMC1 | 1.34 |
| Q13753 | Laminin subunit gamma-2 | LAMC2 | 0.53 |
| O43813 | LanC-like protein 1 | LANCL1 | MYOF |
| Q01650 | Large neutral amino acids transporter small subunit 1 | SLC7A5 | 1.25 |
| Q08722 | Leukocyte surface antigen CD47 | CD47 | 0.43 |
| Q86X29 | Lipolysis-stimulated lipoprotein receptor | LSR | 0.91 |
| P00338 | L-lactate dehydrogenase A chain | LDHA | 0.79 |
| P07195 | L-lactate dehydrogenase B chain | LDHB | 1.20 |
| Q7Z4F1 | Low-density lipoprotein receptor-related protein 10 | LRP10 | 1.73 |
| P11279 | Lysosome-associated membrane glycoprotein 1 | LAMP1 | 4.56 |
| Q14764 | Major vault protein | MVP | 1.41 |
| P40926 | Malate dehydrogenase, mitochondrial | MDH2 | MYOF |
| P50281 | Matrix metalloproteinase-14 | MMP14 | 0.87 |
| Q8N4C8 | Misshapen-like kinase 1 | MINK1 | 0.85 |
| Q8WV92 | MIT domain-containing protein 1 | MITD1 | 0.84 |
| O95819 | Mitogen-activated protein kinase kinase kinase kinase 4 | MAP4K4 | 0.83 |
| P26038 | Moesin | MSN | 0.82 |
| P53985 | Monocarboxylate transporter 1 | SLC16A1 | 1.21 |
| O15427 | Monocarboxylate transporter 4 | SLC16A3 | 1.25 |
| Q99102 | Mucin-4 | MUC4 | 3.01 |
| P33527 | Multidrug resistance-associated protein 1 | ABCC1 | MYOF |
| Q96EY5 | Multivesicular body subunit 12A | MVB12A | 1.09 |
| O95297 | Myelin protein zero-like protein 1 | MPZL1 | 0.74 |
| O60487 | Myelin protein zero-like protein 2 | MPZL2 | 1.26 |
| Q9NZM1 | Myoferlin | MYOF | 11.68 |
| O14745 | Na(+)/H(+) exchange regulatory cofactor NHE-RF1 | SLC9A3R1 | 1.02 |
| P15559 | NAD(P)H dehydrogenase [quinone] 1 | NQO1 | 0.71 |
| Q9Y2A7 | Nck-associated protein 1 | NCKAP1 | 0.75 |
| Q15223 | Nectin-1 | PVRL1 | 1.54 |
| Q92692 | Nectin-2 | PVRL2 | 1.09 |
| Q9BT67 | NEDD4 family-interacting protein 1 | NDFIP1 | 3.46 |
| Q9NV92 | NEDD4 family-interacting protein 2 | NDFIP2 | CTRL |
| Q09666 | Neuroblast differentiation-associated protein AHNAK | AHNAK | 0.61 |
| Q14697 | Neutral alpha-glucosidase AB | GANAB | 0.66 |
| Q15758 | Neutral amino acid transporter B(0) | SLC1A5 | 1.46 |
| Q96TA1 | Niban-like protein 1 | FAM129B | MYOF |
| Q92542 | Nicastrin | NCSTN | 1.49 |
| P43490 | Nicotinamide phosphoribosyltransferase | NAMPT | MYOF |
| O15118 | Niemann-Pick C1 protein | NPC1 | 5.71 |
| P06748 | Nucleophosmin | NPM1 | 0.30 |
| Q9NTK5 | Obg-like ATPase 1 | OLA1 | CTRL |
| Q16625 | Occludin | OCLN | 1.12 |
| Q99650 | Oncostatin-M-specific receptor subunit beta | OSMR | 1.68 |
| Q9C0B5 | Palmitoyltransferase ZDHHC5 | ZDHHC5 | 0.87 |
| P62937 | Peptidyl-prolyl cis-trans isomerase A | PPIA | 1.04 |
| O14936 | Peripheral plasma membrane protein CASK | CASK | 0.74 |
| Q06830 | Peroxiredoxin-1 | PRDX1 | 0.86 |
| P30044 | Peroxiredoxin-5, mitochondrial | PRDX5 | 0.92 |
| P30041 | Peroxiredoxin-6 | PRDX6 | 1.00 |
| Q9BTU6 | Phosphatidylinositol 4-kinase type 2-alpha | PI4K2A | CTRL |
| P00558 | Phosphoglycerate kinase 1 | PGK1 | 0.74 |
| P18669 | Phosphoglycerate mutase 1 | PGAM1 | 1.10 |
| P36969 | Phospholipid hydroperoxide glutathione peroxidase, mitochondrial | GPX4 | MYOF |
| O15162 | Phospholipid scramblase 1 | PLSCR1 | 1.27 |
| Q9NRY6 | Phospholipid scramblase 3 | PLSCR3 | 0.95 |
| Q9Y446 | Plakophilin-3 | PKP3 | 0.90 |
| Q99569 | Plakophilin-4 | PKP4 | 0.41 |
| P05120 | Plasminogen activator inhibitor 2 | SERPINB2 | 0.16 |
| P13796 | Plastin-2 | LCP1 | 1.48 |
| P13797 | Plastin-3 | PLS3 | 0.32 |
| Q15149 | Plectin | PLEC | 0.59 |
| Q9UIW2 | Plexin-A1 | PLXNA1 | 1.10 |
| O15031 | Plexin-B2 | PLXNB2 | 0.93 |
| Q9UKK3 | Poly [ADP-ribose] polymerase 4 | PARP4 | 2.05 |
| Q15365 | Poly(rC)-binding protein 1 | PCBP1 | 0.63 |
| Q15366 | Poly(rC)-binding protein 2 | PCBP2 | MYOF |
| P11940 | Polyadenylate-binding protein 1 | PABPC1 | 0.63 |
| P0CG47 | Polyubiquitin-B | UBB | 0.86 |
| P02545 | Prelamin-A/C | LMNA | 1.33 |
| Q5W0Z9 | Probable palmitoyltransferase ZDHHC20 | ZDHHC20 | 1.37 |
| Q9H3G5 | Probable serine carboxypeptidase CPVL | CPVL | CTRL |
| P09668 | Pro-cathepsin H | CTSH | 0.44 |
| Q02809 | Procollagen-lysine,2-oxoglutarate 5-dioxygenase 1 | PLOD1 | MYOF |
| P07737 | Profilin-1 | PFN1 | 0.85 |
| Q8WUM4 | Programmed cell death 6-interacting protein | PDCD6IP | 1.18 |
| O75340 | Programmed cell death protein 6 | PDCD6 | 1.03 |
| P12004 | Proliferating cell nuclear antigen | PCNA | MYOF |
| Q9UQ80 | Proliferation-associated protein 2G4 | PA2G4 | 0.14 |
| Q07954 | Prolow-density lipoprotein receptor-related protein 1 | LRP1 | 0.98 |
| Q8N271 | Prominin-2 | PROM2 | 1.37 |
| Q8NBP7 | Proprotein convertase subtilisin/kexin type 9 | PCSK9 | 1.35 |
| Q9P2B2 | Prostaglandin F2 receptor negative regulator | PTGFRN | 0.89 |
| P28072 | Proteasome subunit beta type-6 | PSMB6 | 2.55 |
| Q99873 | Protein arginine N-methyltransferase 1 | PRMT1 | 0.43 |
| P07237 | Protein disulfide-isomerase | P4HB | 1.60 |
| P30101 | Protein disulfide-isomerase A3 | PDIA3 | 1.19 |
| Q92520 | Protein FAM3C | FAM3C | 1.60 |
| P78504 | Protein jagged-1 | JAG1 | 1.07 |
| Q9UNF0 | Protein kinase C and casein kinase substrate in neurons protein 2 | PACSIN2 | 1.08 |
| Q9UKS6 | Protein kinase C and casein kinase substrate in neurons protein 3 | PACSIN3 | 1.23 |
| Q92597 | Protein NDRG1 | NDRG1 | 0.39 |
| Q9UGV2 | Protein NDRG3 | NDRG3 | MYOF |
| P60903 | Protein S100-A10 | S100A10 | 4.45 |
| Q92734 | Protein TFG | TFG | 0.90 |
| Q9C0H2 | Protein tweety homolog 3 | TTYH3 | 1.59 |
| Q9GZT5 | Protein Wnt-10a | WNT10A | CTRL |
| O75695 | Protein XRP2 | RP2 | 1.58 |
| P21980 | Protein-glutamine gamma-glutamyltransferase 2 | TGM2 | 1.20 |
| Q14517 | Protocadherin Fat 1 | FAT1 | 0.49 |
| Q9UN70 | Protocadherin gamma-C3 | PCDHGC3 | 1.52 |
| P12931 | Proto-oncogene tyrosine-protein kinase Src | SRC | 0.89 |
| O43865 | Putative adenosylhomocysteinase 2 | AHCYL1 | MYOF |
| Q5VTE0 | Putative elongation factor 1-alpha-like 3 | EEF1A1P5 | 0.84 |
| O43143 | Putative pre-mRNA-splicing factor ATP-dependent RNA helicase DHX15 | DHX15 | MYOF |
| P14618 | Pyruvate kinase PKM | PKM | 0.50 |
| P50395 | Rab GDP dissociation inhibitor beta | GDI2 | 1.02 |
| Q9H0H5 | Rac GTPase-activating protein 1 | RACGAP1 | 1.32 |
| P35241 | Radixin | RDX | 2.10 |
| P46940 | Ras GTPase-activating-like protein IQGAP1 | IQGAP1 | 0.55 |
| Q15404 | Ras suppressor protein 1 | RSU1 | 1.21 |
| P63000 | Ras-related C3 botulinum toxin substrate 1 | RAC1 | 1.04 |
| P61026 | Ras-related protein Rab-10 | RAB10 | 0.91 |
| Q15907 | Ras-related protein Rab-11B | RAB11B | 1.02 |
| P61106 | Ras-related protein Rab-14 | RAB14 | 1.23 |
| P62820 | Ras-related protein Rab-1A | RAB1A | 1.03 |
| Q9H0U4 | Ras-related protein Rab-1B | RAB1B | 1.43 |
| Q9UL25 | Ras-related protein Rab-21 | RAB21 | 1.39 |
| P61019 | Ras-related protein Rab-2A | RAB2A | 1.44 |
| Q15286 | Ras-related protein Rab-35 | RAB35 | 0.93 |
| P61020 | Ras-related protein Rab-5B | RAB5B | 1.23 |
| P51148 | Ras-related protein Rab-5C | RAB5C | 1.30 |
| P51149 | Ras-related protein Rab-7a | RAB7A | 1.59 |
| P61006 | Ras-related protein Rab-8A | RAB8A | 1.08 |
| P11234 | Ras-related protein Ral-B | RALB | 0.98 |
| P61224 | Ras-related protein Rap-1b | RAP1B | 0.79 |
| P61225 | Ras-related protein Rap-2b | RAP2B | 0.86 |
| Q9Y3L5 | Ras-related protein Rap-2c | RAP2C | 0.46 |
| P10301 | Ras-related protein R-Ras | RRAS | 0.83 |
| P18433 | Receptor-type tyrosine-protein phosphatase alpha | PTPRA | 0.94 |
| P23469 | Receptor-type tyrosine-protein phosphatase epsilon | PTPRE | 1.29 |
| P10586 | Receptor-type tyrosine-protein phosphatase F | PTPRF | 1.54 |
| Q8NFJ5 | Retinoic acid-induced protein 3 | GPRC5A | 0.94 |
| Q9Y265 | RuvB-like 1 | RUVBL1 | 0.69 |
| Q9Y230 | RuvB-like 2 | RUVBL2 | 0.68 |
| O14828 | Secretory carrier-associated membrane protein 3 | SCAMP3 | 2.25 |
| Q12884 | Seprase | FAP | CTRL |
| Q13501 | Sequestosome-1 | SQSTM1 | 2.51 |
| Q86VE9 | Serine incorporator 5 | SERINC5 | 0.91 |
| O15269 | Serine palmitoyltransferase 1 | SPTLC1 | 1.52 |
| O95084 | Serine protease 23 | PRSS23 | 1.01 |
| Q92743 | Serine protease HTRA1 | HTRA1 | 3.01 |
| Q7KZI7 | Serine/threonine-protein kinase MARK2 | MARK2 | MYOF |
| P30153 | Serine/threonine-protein phosphatase 2A 65 kDa regulatory subunit A alpha isoform | PPP2R1A | 0.54 |
| P62136 | Serine/threonine-protein phosphatase PP1-alpha catalytic subunit | PPP1CA | 0.72 |
| P62318 | Small nuclear ribonucleoprotein Sm D3 | SNRPD3 | MYOF |
| Q8NCG7 | Sn1-specific diacylglycerol lipase beta | DAGLB | CTRL |
| P19634 | Sodium/hydrogen exchanger 1 | SLC9A1 | MYOF |
| P05023 | Sodium/potassium-transporting ATPase subunit alpha-1 | ATP1A1 | 0.95 |
| P05026 | Sodium/potassium-transporting ATPase subunit beta-1 | ATP1B1 | 0.45 |
| P54709 | Sodium/potassium-transporting ATPase subunit beta-3 | ATP1B3 | 2.25 |
| Q9H2H9 | Sodium-coupled neutral amino acid transporter 1 | SLC38A1 | 0.70 |
| Q96QD8 | Sodium-coupled neutral amino acid transporter 2 | SLC38A2 | 0.52 |
| Q8WUX1 | Sodium-coupled neutral amino acid transporter 5 | SLC38A5 | 0.85 |
| Q08357 | Sodium-dependent phosphate transporter 2 | SLC20A2 | 2.11 |
| P55011 | Solute carrier family 12 member 2 | SLC12A2 | 1.24 |
| Q9UHW9 | Solute carrier family 12 member 6 | SLC12A6 | 1.19 |
| P11166 | Solute carrier family 2, facilitated glucose transporter member 1 | SLC2A1 | 0.76 |
| P30626 | Sorcin | SRI | 1.16 |
| Q9UMY4 | Sorting nexin-12 | SNX12 | 2.37 |
| Q15036 | Sorting nexin-17 | SNX17 | CTRL |
| O60493 | Sorting nexin-3 | SNX3 | 1.34 |
| P38646 | Stress-70 protein, mitochondrial | HSPA9 | MYOF |
| P31948 | Stress-induced-phosphoprotein 1 | STIP1 | 0.91 |
| Q8TED4 | Sugar phosphate exchanger 2 | SLC37A2 | CTRL |
| Q9Y5Y6 | Suppressor of tumorigenicity 14 protein | ST14 | 1.33 |
| O60687 | Sushi repeat-containing protein SRPX2 | SRPX2 | 0.79 |
| O43760 | Synaptogyrin-2 | SYNGR2 | 1.65 |
| O43752 | Syntaxin-6 | STX6 | 1.79 |
| O00186 | Syntaxin-binding protein 3 | STXBP3 | 1.23 |
| O00560 | Syntenin-1 | SDCBP | 0.96 |
| Q9H190 | Syntenin-2 | SDCBP2 | 1.06 |
| Q9Y490 | Talin-1 | TLN1 | 0.94 |
| Q86VP1 | Tax1-binding protein 1 | TAX1BP1 | 2.09 |
| P17987 | T-complex protein 1 subunit alpha | TCP1 | 0.60 |
| P78371 | T-complex protein 1 subunit beta | CCT2 | 0.60 |
| P50991 | T-complex protein 1 subunit delta | CCT4 | 0.55 |
| P48643 | T-complex protein 1 subunit epsilon | CCT5 | 0.44 |
| Q99832 | T-complex protein 1 subunit eta | CCT7 | 0.60 |
| P49368 | T-complex protein 1 subunit gamma | CCT3 | 0.88 |
| P50990 | T-complex protein 1 subunit theta | CCT8 | 0.67 |
| P40227 | T-complex protein 1 subunit zeta | CCT6A | 0.65 |
| Q8NG11 | Tetraspanin-14 | TSPAN14 | 0.83 |
| O95858 | Tetraspanin-15 | TSPAN15 | CTRL |
| O43657 | Tetraspanin-6 | TSPAN6 | 1.30 |
| P10599 | Thioredoxin | TXN | 1.29 |
| Q9H3M7 | Thioredoxin-interacting protein | TXNIP | 0.81 |
| P26639 | Threonine--tRNA ligase, cytoplasmic | TARS | 0.46 |
| P07204 | Thrombomodulin | THBD | 0.36 |
| Q07157 | Tight junction protein ZO-1 | TJP1 | 1.10 |
| P00750 | Tissue-type plasminogen activator | PLAT | 3.70 |
| Q15025 | TNFAIP3-interacting protein 1 | TNIP1 | 1.04 |
| Q9H0E2 | Toll-interacting protein | TOLLIP | 1.11 |
| P02786 | Transferrin receptor protein 1 | TFRC | 1.69 |
| Q15582 | Transforming growth factor-beta-induced protein ig-h3 | TGFBI | 0.10 |
| P61586 | Transforming protein RhoA | RHOA | 0.71 |
| P37802 | Transgelin-2 | TAGLN2 | 1.06 |
| P55072 | Transitional endoplasmic reticulum ATPase | VCP | 1.36 |
| P29401 | Transketolase | TKT | 1.87 |
| P13693 | Translationally-controlled tumor protein | TPT1 | 1.23 |
| Q7Z403 | Transmembrane channel-like protein 6 | TMC6 | 0.42 |
| Q9BXS4 | Transmembrane protein 59 | TMEM59 | 1.51 |
| P60174 | Triosephosphate isomerase | TPI1 | 0.87 |
| P29144 | Tripeptidyl-peptidase 2 | TPP2 | 2.95 |
| Q9BQE3 | Tubulin alpha-1C chain | TUBA1C | 0.75 |
| P68366 | Tubulin alpha-4A chain | TUBA4A | 0.91 |
| P07437 | Tubulin beta chain | TUBB | 0.66 |
| P68371 | Tubulin beta-4B chain | TUBB4B | 0.79 |
| Q14166 | Tubulin--tyrosine ligase-like protein 12 | TTLL12 | MYOF |
| P21580 | Tumor necrosis factor alpha-induced protein 3 | TNFAIP3 | 0.59 |
| O75509 | Tumor necrosis factor receptor superfamily member 21 | TNFRSF21 | 2.13 |
| Q99816 | Tumor susceptibility gene 101 protein | TSG101 | 1.36 |
| P09758 | Tumor-associated calcium signal transducer 2 | TACSTD2 | 0.95 |
| P23458 | Tyrosine-protein kinase JAK1 | JAK1 | 2.10 |
| P07947 | Tyrosine-protein kinase Yes | YES1 | 1.16 |
| O75643 | U5 small nuclear ribonucleoprotein 200 kDa helicase | SNRNP200 | MYOF |
| P61088 | Ubiquitin-conjugating enzyme E2 N | UBE2N | 0.37 |
| Q8IX04 | Ubiquitin-conjugating enzyme E2 variant 3 | UEVLD | 0.64 |
| P22314 | Ubiquitin-like modifier-activating enzyme 1 | UBA1 | 0.53 |
| O60701 | UDP-glucose 6-dehydrogenase | UGDH | 1.39 |
| Q9P206 | Uncharacterized protein KIAA1522 | KIAA1522 | MYOF |
| O43795 | Unconventional myosin-Ib | MYO1B | 0.85 |
| O00159 | Unconventional myosin-Ic | MYO1C | 0.91 |
| O94832 | Unconventional myosin-Id | MYO1D | 1.19 |
| P00749 | Urokinase-type plasminogen activator | PLAU | 0.86 |
| Q16851 | UTP--glucose-1-phosphate uridylyltransferase | UGP2 | 0.53 |
| O75436 | Vacuolar protein sorting-associated protein 26A | VPS26A | MYOF |
| Q9UK41 | Vacuolar protein sorting-associated protein 28 homolog | VPS28 | 1.19 |
| Q96QK1 | Vacuolar protein sorting-associated protein 35 | VPS35 | 0.82 |
| Q9H9H4 | Vacuolar protein sorting-associated protein 37B | VPS37B | 0.89 |
| A5D8V6 | Vacuolar protein sorting-associated protein 37C | VPS37C | 2.25 |
| Q9UN37 | Vacuolar protein sorting-associated protein 4A | VPS4A | 0.88 |
| O75351 | Vacuolar protein sorting-associated protein 4B | VPS4B | 0.96 |
| P50552 | Vasodilator-stimulated phosphoprotein | VASP | 2.03 |
| Q9UEU0 | Vesicle transport through interaction with t-SNAREs homolog 1B | VTI1B | 2.59 |
| P51809 | Vesicle-associated membrane protein 7 | VAMP7 | 1.88 |
| P18206 | Vinculin | VCL | 1.05 |
| Q8IWT6 | Volume-regulated anion channel subunit LRRC8A | LRRC8A | 1.31 |
| O75083 | WD repeat-containing protein 1 | WDR1 | 0.52 |
| Q9Y6W5 | Wiskott-Aldrich syndrome protein family member 2 | WASF2 | MYOF |
| P13010 | X-ray repair cross-complementing protein 5 | XRCC5 | MYOF |
| P12956 | X-ray repair cross-complementing protein 6 | XRCC6 | 0.81 |
