# Supplementary material for: Myoferlin is a novel exosomal protein and functional regulator of cancer-derived exosomes
Source: Oncotarget. 2016 Nov 10;7(50):83669–83. doi: 10.18632/oncotarget.13276 (PMC5347796; doi:10.18632/oncotarget.13276)
Supplement: Supplementary file 3 [file oncotarget-07-83669-s003.docx]

**Supplementary Table S2**

**Modulated proteins in exosomes isolated from MDA-MB-231.**

| **Accession** | **Protein Description** | **CTRL/MYOF Ratio** |
| --- | --- | --- |
| P61981 | 14-3-3 protein gamma | CTRL |
| P31947 | 14-3-3 protein sigma | 0.62 |
| P62333 | 26S protease regulatory subunit 10B | 3.01 |
| P62191 | 26S protease regulatory subunit 4 | CTRL |
| P17980 | 26S protease regulatory subunit 6A | CTRL |
| P43686 | 26S protease regulatory subunit 6B | 4.51 |
| P35998 | 26S protease regulatory subunit 7 | 2.49 |
| P62195 | 26S protease regulatory subunit 8 | CTRL |
| Q99460 | 26S proteasome non-ATPase regulatory subunit 1 | CTRL |
| Q13200 | 26S proteasome non-ATPase regulatory subunit 2 | 1.78 |
| P48556 | 26S proteasome non-ATPase regulatory subunit 8 | 3.68 |
| P62277 | 40S ribosomal protein S13 | CTRL |
| P62249 | 40S ribosomal protein S16 | CTRL |
| P15880 | 40S ribosomal protein S2 | CTRL |
| P61247 | 40S ribosomal protein S3a | CTRL |
| P62701 | 40S ribosomal protein S4 | CTRL |
| P62241 | 40S ribosomal protein S8 | CTRL |
| P46781 | 40S ribosomal protein S9 | 3.31 |
| P08865 | 40S ribosomal protein SA | CTRL |
| P50914 | 60S ribosomal protein L14 | CTRL |
| P61313 | 60S ribosomal protein L15 | 2.65 |
| P18621 | 60S ribosomal protein L17 | CTRL |
| P83731 | 60S ribosomal protein L24 | CTRL |
| P36578 | 60S ribosomal protein L4 | 2.62 |
| P46777 | 60S ribosomal protein L5 | CTRL |
| Q02878 | 60S ribosomal protein L6 | 5.92 |
| P18124 | 60S ribosomal protein L7 | CTRL |
| P62424 | 60S ribosomal protein L7a | 5.21 |
| P11021 | 78 kDa glucose-regulated protein | 2.56 |
| P61158 | Actin-related protein 3 | CTRL |
| P84077 | ADP-ribosylation factor 1 | 1.78 |
| P18085 | ADP-ribosylation factor 4 | 1.71 |
| P62330 | ADP-ribosylation factor 6 | 1.91 |
| Q9NVJ2 | ADP-ribosylation factor-like protein 8B | 8.22 |
| O00468 | Agrin | 2.83 |
| P14550 | Alcohol dehydrogenase [NADP(+)] | 0.65 |
| O43707 | Alpha-actinin-4 | 1.51 |
| P12429 | Annexin A3 | CTRL |
| P20073 | Annexin A7 | CTRL |
| P63010 | AP-2 complex subunit beta | 2.64 |
| P04114 | Apolipoprotein B-100 | 0.59 |
| P00966 | Argininosuccinate synthase | 0.41 |
| Q8N5I2 | Arrestin domain-containing protein 1 | CTRL |
| P53396 | ATP-citrate synthase | 1.98 |
| Q01813 | ATP-dependent 6-phosphofructokinase, platelet type | 2.60 |
| O00148 | ATP-dependent RNA helicase DDX39A | CTRL |
| O00571 | ATP-dependent RNA helicase DDX3X | CTRL |
| P61769 | Beta-2-microglobulin | 0.55 |
| P07814 | Bifunctional glutamate/proline--tRNA ligase | 4.63 |
| Q9UQB8 | Brain-specific angiogenesis inhibitor 1-associated protein 2 | CTRL |
| Q5VW32 | BRO1 domain-containing protein BROX | CTRL |
| O15484 | Calpain-5 | CTRL |
| Q8NEV1 | Casein kinase II subunit alpha 3 | CTRL |
| P35222 | Catenin beta-1 | 3.62 |
| Q03135 | Caveolin-1 | 2.17 |
| Q9Y5K6 | CD2-associated protein | CTRL |
| P08962 | CD63 antigen | CTRL |
| Q16739 | Ceramide glucosyltransferase | CTRL |
| O43633 | Charged multivesicular body protein 2a | CTRL |
| Q8IWA5 | Choline transporter-like protein 2 | 3.68 |
| Q00610 | Clathrin heavy chain 1 | 2.59 |
| P09497 | Clathrin light chain B | CTRL |
| P20908 | Collagen alpha-1(V) chain | 1.79 |
| P39060 | Collagen alpha-1(XVIII) chain | 1.72 |
| Q99829 | Copine-1 | CTRL |
| O75131 | Copine-3 | 2.15 |
| Q86YQ8 | Copine-8 | 3.29 |
| P06493 | Cyclin-dependent kinase 1 | 3.00 |
| Q14204 | Cytoplasmic dynein 1 heavy chain 1 | 3.08 |
| O00154 | Cytosolic acyl coenzyme A thioester hydrolase | CTRL |
| Q14126 | Desmoglein-2 | 3.16 |
| P15924 | Desmoplakin | 0.54 |
| Q9P265 | Disco-interacting protein 2 homolog B | 2.26 |
| P11387 | DNA topoisomerase 1 | CTRL |
| P78527 | DNA-dependent protein kinase catalytic subunit | CTRL |
| O60884 | DnaJ homolog subfamily A member 2 | 2.82 |
| P25685 | DnaJ homolog subfamily B member 1 | 2.19 |
| P50570 | Dynamin-2 | 3.98 |
| Q14258 | E3 ubiquitin/ISG15 ligase TRIM25 | CTRL |
| P22413 | Ectonucleotide pyrophosphatase/phosphodiesterase family member 1 | 0.63 |
| O43854 | EGF-like repeat and discoidin I-like domain-containing protein 3 | 1.54 |
| Q9NZN4 | EH domain-containing protein 2 | 2.20 |
| P26641 | Elongation factor 1-gamma | 2.55 |
| P14625 | Endoplasmin | CTRL |
| P38919 | Eukaryotic initiation factor 4A-III | CTRL |
| P63241 | Eukaryotic translation initiation factor 5A-1 | 2.93 |
| P56537 | Eukaryotic translation initiation factor 6 | CTRL |
| P49327 | Fatty acid synthase | 1.78 |
| P02794 | Ferritin heavy chain | 2.63 |
| P02792 | Ferritin light chain | 1.97 |
| O75369 | Filamin-B | 8.50 |
| O75955 | Flotillin-1 | 4.22 |
| Q14254 | Flotillin-2 | 4.25 |
| P11413 | Glucose-6-phosphate 1-dehydrogenase | 5.14 |
| Q06210 | Glutamine--fructose-6-phosphate aminotransferase [isomerizing] 1 | 2.94 |
| Q7RTV2 | Glutathione S-transferase A5 | 0.54 |
| P62993 | Growth factor receptor-bound protein 2 | CTRL |
| Q15382 | GTP-binding protein Rheb | 2.32 |
| P63244 | Guanine nucleotide-binding protein subunit beta-2-like 1 | CTRL |
| P08107 | Heat shock 70 kDa protein 1A/1B | 1.59 |
| Q92598 | Heat shock protein 105 kDa | 2.69 |
| P26927 | Hepatocyte growth factor-like protein | MYOF |
| P61978 | Heterogeneous nuclear ribonucleoprotein K | CTRL |
| P52272 | Heterogeneous nuclear ribonucleoprotein M | CTRL |
| P30825 | High affinity cationic amino acid transporter 1 | 2.37 |
| P62805 | Histone H4 | 1.88 |
| P01892 | HLA class I histocompatibility antigen, A-2 alpha chain | 1.74 |
| Q969P0 | Immunoglobulin superfamily member 8 | CTRL |
| P12268 | Inosine-5-monophosphate dehydrogenase 2 | 3.36 |
| P06756 | Integrin alpha-V | 1.52 |
| P05106 | Integrin beta-3 | 1.68 |
| O75874 | Isocitrate dehydrogenase [NADP] cytoplasmic | CTRL |
| P41252 | Isoleucine--tRNA ligase, cytoplasmic | CTRL |
| P53990 | IST1 homolog | 1.52 |
| P14923 | Junction plakoglobin | 0.67 |
| Q08431 | Lactadherin | 2.12 |
| Q9P2J5 | Leucine--tRNA ligase, cytoplasmic | CTRL |
| Q86X29 | Lipolysis-stimulated lipoprotein receptor | 3.77 |
| O60488 | Long-chain-fatty-acid--CoA ligase 4 | 2.14 |
| Q7Z4F1 | Low-density lipoprotein receptor-related protein 10 | CTRL |
| Q9H3U5 | Major facilitator superfamily domain-containing protein 1 | CTRL |
| Q14764 | Major vault protein | 2.18 |
| P20774 | Mimecan | 0.58 |
| O95297 | Myelin protein zero-like protein 1 | 0.27 |
| Q96S97 | Myeloid-associated differentiation marker | CTRL |
| Q9NZM1 | Myoferlin | CTRL |
| Q9Y2A7 | Nck-associated protein 1 | CTRL |
| Q15758 | Neutral amino acid transporter B(0) | 1.84 |
| Q96TA1 | Niban-like protein 1 | MYOF |
| P43490 | Nicotinamide phosphoribosyltransferase | 2.53 |
| O15118 | Niemann-Pick C1 protein | 3.85 |
| Q99650 | Oncostatin-M-specific receptor subunit beta | CTRL |
| Q9C0B5 | Palmitoyltransferase ZDHHC5 | CTRL |
| Q9BTU6 | Phosphatidylinositol 4-kinase type 2-alpha | CTRL |
| P05121 | Plasminogen activator inhibitor 1 | CTRL |
| Q15149 | Plectin | CTRL |
| Q9UKK3 | Poly [ADP-ribose] polymerase 4 | 4.87 |
| P11940 | Polyadenylate-binding protein 1 | CTRL |
| P0CG47 | Polyubiquitin-B | 1.62 |
| O75340 | Programmed cell death protein 6 | 2.18 |
| O00622 | Protein CYR61 | 3.88 |
| P07237 | Protein disulfide-isomerase | CTRL |
| Q92734 | Protein TFG | CTRL |
| Q9C0H2 | Protein tweety homolog 3 | 2.15 |
| P12931 | Proto-oncogene tyrosine-protein kinase Src | 2.35 |
| Q9Y315 | Putative deoxyribose-phosphate aldolase | CTRL |
| O43143 | Putative pre-mRNA-splicing factor ATP-dependent RNA helicase DHX15 | CTRL |
| P50395 | Rab GDP dissociation inhibitor beta | 1.83 |
| Q14699 | Raftlin | 2.35 |
| P46940 | Ras GTPase-activating-like protein IQGAP1 | 3.04 |
| Q15404 | Ras suppressor protein 1 | 0.50 |
| P15153 | Ras-related C3 botulinum toxin substrate 2 | 3.12 |
| P10301 | Ras-related protein R-Ras | 1.92 |
| P62070 | Ras-related protein R-Ras2 | 2.29 |
| Q9UL26 | Ras-related protein Rab-22A | 4.12 |
| P61019 | Ras-related protein Rab-2A | CTRL |
| P61020 | Ras-related protein Rab-5B | 2.33 |
| P51148 | Ras-related protein Rab-5C | 1.74 |
| P51149 | Ras-related protein Rab-7a | 1.55 |
| P18433 | Receptor-type tyrosine-protein phosphatase alpha | CTRL |
| P10586 | Receptor-type tyrosine-protein phosphatase F | 3.97 |
| P23470 | Receptor-type tyrosine-protein phosphatase gamma | CTRL |
| Q15262 | Receptor-type tyrosine-protein phosphatase kappa | 4.22 |
| Q8NFJ5 | Retinoic acid-induced protein 3 | 4.24 |
| Q9Y265 | RuvB-like 1 | 4.07 |
| Q9Y230 | RuvB-like 2 | CTRL |
| O14828 | Secretory carrier-associated membrane protein 3 | 3.23 |
| Q13501 | Sequestosome-1 | CTRL |
| O95747 | Serine/threonine-protein kinase OSR1 | 4.35 |
| Q92783 | Signal transducing adapter molecule 1 | 3.98 |
| P62318 | Small nuclear ribonucleoprotein Sm D3 | CTRL |
| Q8NCG7 | Sn1-specific diacylglycerol lipase beta | CTRL |
| Q9H2H9 | Sodium-coupled neutral amino acid transporter 1 | 1.50 |
| P54709 | Sodium/potassium-transporting ATPase subunit beta-3 | CTRL |
| P55011 | Solute carrier family 12 member 2 | 2.76 |
| P11166 | Solute carrier family 2, facilitated glucose transporter member 1 | 1.71 |
| Q15036 | Sorting nexin-17 | CTRL |
| O60493 | Sorting nexin-3 | 3.65 |
| Q6UWP8 | Suprabasin | 0.65 |
| O43752 | Syntaxin-6 | CTRL |
| O00560 | Syntenin-1 | 1.80 |
| P48643 | T-complex protein 1 subunit epsilon | 2.08 |
| Q99832 | T-complex protein 1 subunit eta | CTRL |
| Q86VP1 | Tax1-binding protein 1 | CTRL |
| Q9P273 | Teneurin-3 | CTRL |
| Q8NG11 | Tetraspanin-14 | 4.47 |
| P37173 | TGF-beta receptor type-2 | CTRL |
| P10599 | Thioredoxin | CTRL |
| Q9H3M7 | Thioredoxin-interacting protein | CTRL |
| P13726 | Tissue factor | CTRL |
| P02786 | Transferrin receptor protein 1 | 3.02 |
| Q15582 | Transforming growth factor-beta-induced protein ig-h3 | 0.36 |
| Q92616 | Translational activator GCN1 | CTRL |
| P29144 | Tripeptidyl-peptidase 2 | 2.77 |
| Q9BQE3 | Tubulin alpha-1C chain | 1.99 |
| P07437 | Tubulin beta chain | 1.82 |
| P68371 | Tubulin beta-4B chain | 1.59 |
| Q99816 | Tumor susceptibility gene 101 protein | 5.07 |
| P23458 | Tyrosine-protein kinase JAK1 | CTRL |
| P30530 | Tyrosine-protein kinase receptor UFO | 2.61 |
| P07947 | Tyrosine-protein kinase Yes | CTRL |
| P09012 | U1 small nuclear ribonucleoprotein A | MYOF |
| A0AVT1 | Ubiquitin-like modifier-activating enzyme 6 | CTRL |
| O60701 | UDP-glucose 6-dehydrogenase | CTRL |
| O00159 | Unconventional myosin-Ic | 2.69 |
| P00749 | Urokinase-type plasminogen activator | 0.66 |
| Q9UK41 | Vacuolar protein sorting-associated protein 28 homolog | 6.45 |
| Q9H9H4 | Vacuolar protein sorting-associated protein 37B | CTRL |
| O75351 | Vacuolar protein sorting-associated protein 4B | 6.04 |
| P51809 | Vesicle-associated membrane protein 7 | CTRL |
| P13010 | X-ray repair cross-complementing protein 5 | CTRL |
| P12956 | X-ray repair cross-complementing protein 6 | 2.50 |

**Modulated proteins in exosomes isolated from BxPC3.**

| **Accession** | **Protein Description** | **CTRL/MYOF Ratio** |
| --- | --- | --- |
| P62191 | 26S protease regulatory subunit 4 | CTRL |
| P43686 | 26S protease regulatory subunit 6B | 0.43 |
| O00232 | 26S proteasome non-ATPase regulatory subunit 12 | 0.37 |
| Q9UNM6 | 26S proteasome non-ATPase regulatory subunit 13 | 0.49 |
| P48556 | 26S proteasome non-ATPase regulatory subunit 8 | 0.54 |
| P25398 | 40S ribosomal protein S12 | MYOF |
| P62277 | 40S ribosomal protein S13 | MYOF |
| P62249 | 40S ribosomal protein S16 | 0.51 |
| P60866 | 40S ribosomal protein S20 | 0.23 |
| P23396 | 40S ribosomal protein S3 | 0.23 |
| P61247 | 40S ribosomal protein S3a | 0.38 |
| P62701 | 40S ribosomal protein S4, X isoform | 0.40 |
| P08865 | 40S ribosomal protein SA | 0.32 |
| P52209 | 6-phosphogluconate dehydrogenase, decarboxylating | 0.57 |
| P05388 | 60S acidic ribosomal protein P0 | MYOF |
| P27635 | 60S ribosomal protein L10 | 0.41 |
| P62906 | 60S ribosomal protein L10a | MYOF |
| P18621 | 60S ribosomal protein L17 | 0.52 |
| Q02878 | 60S ribosomal protein L6 | 0.35 |
| P11021 | 78 kDa glucose-regulated protein | 3.66 |
| P61160 | Actin-related protein 2 | 0.52 |
| O15144 | Actin-related protein 2/3 complex subunit 2 | 0.42 |
| O15145 | Actin-related protein 2/3 complex subunit 3 | 0.49 |
| P61158 | Actin-related protein 3 | 0.37 |
| P55263 | Adenosine kinase | 0.42 |
| Q9NVJ2 | ADP-ribosylation factor-like protein 8B | 1.85 |
| P11766 | Alcohol dehydrogenase class-3 | MYOF |
| P47895 | Aldehyde dehydrogenase family 1 member A3 | 0.61 |
| Q04828 | Aldo-keto reductase family 1 member C1 | 0.38 |
| O43707 | Alpha-actinin-4 | 1.98 |
| P50995 | Annexin A11 | 0.63 |
| P20073 | Annexin A7 | 0.64 |
| P04424 | Argininosuccinate lyase | MYOF |
| P08243 | Asparagine synthetase [glutamine-hydrolyzing] | CTRL |
| P14868 | Aspartate--tRNA ligase, cytoplasmic | 0.36 |
| P06576 | ATP synthase subunit beta, mitochondrial | MYOF |
| P61221 | ATP-binding cassette sub-family E member 1 | MYOF |
| Q01813 | ATP-dependent 6-phosphofructokinase, platelet type | 0.30 |
| Q08211 | ATP-dependent RNA helicase A | 0.22 |
| O00148 | ATP-dependent RNA helicase DDX39A | 0.33 |
| O00571 | ATP-dependent RNA helicase DDX3X | 0.40 |
| Q9H4G0 | Band 4.1-like protein 1 | 1.54 |
| P61769 | Beta-2-microglobulin | 0.20 |
| P07686 | Beta-hexosaminidase subunit beta | 1.80 |
| P07814 | Bifunctional glutamate/proline--tRNA ligase | MYOF |
| P31939 | Bifunctional purine biosynthesis protein PURH | 0.47 |
| P11586 | C-1-tetrahydrofolate synthase, cytoplasmic | 0.45 |
| P10644 | cAMP-dependent protein kinase type I-alpha regulatory subunit | CTRL |
| Q9HCP0 | Casein kinase I isoform gamma-1 | 0.43 |
| Q9Y6M4 | Casein kinase I isoform gamma-3 | MYOF |
| **Q03135** | **Caveolin-1** | 1.77 |
| **P08962** | **CD63 antigen** | 2.29 |
| Q9HD42 | Charged multivesicular body protein 1a | 0.51 |
| O43633 | Charged multivesicular body protein 2a | 0.41 |
| Q9BY43 | Charged multivesicular body protein 4a | 0.52 |
| Q9H444 | Charged multivesicular body protein 4b | 0.48 |
| Q9Y696 | Chloride intracellular channel protein 4 | 2.11 |
| O75390 | Citrate synthase, mitochondrial | MYOF |
| O95832 | Claudin-1 | 0.50 |
| P53618 | Coatomer subunit beta | 0.36 |
| P23528 | Cofilin-1 | 0.65 |
| Q99715 | Collagen alpha-1(XII) chain | 3.49 |
| Q9UMD9 | Collagen alpha-1(XVII) chain | 0.53 |
| P08174 | Complement decay-accelerating factor | MYOF |
| Q12860 | Contactin-1 | 2.16 |
| Q86YQ8 | Copine-8 | 0.58 |
| P06493 | Cyclin-dependent kinase 1 | 0.64 |
| Q9H1C7 | Cysteine-rich and transmembrane domain-containing protein 1 | CTRL |
| Q14204 | Cytoplasmic dynein 1 heavy chain 1 | 0.45 |
| O00154 | Cytosolic acyl coenzyme A thioester hydrolase | MYOF |
| Q13443 | Disintegrin and metalloproteinase domain-containing protein 9 | 0.45 |
| P31689 | DnaJ homolog subfamily A member 1 | 0.33 |
| Q02750 | Dual specificity mitogen-activated protein kinase kinase 1 | 0.29 |
| O95834 | Echinoderm microtubule-associated protein-like 2 | CTRL |
| Q9H223 | EH domain-containing protein 4 | 0.60 |
| P13639 | Elongation factor 2 | 0.48 |
| P27105 | Erythrocyte band 7 integral membrane protein | 0.31 |
| P60842 | Eukaryotic initiation factor 4A-I | 0.57 |
| P38919 | Eukaryotic initiation factor 4A-III | MYOF |
| Q14152 | Eukaryotic translation initiation factor 3 subunit A | MYOF |
| P56537 | Eukaryotic translation initiation factor 6 | MYOF |
| P52907 | F-actin-capping protein subunit alpha-1 | MYOF |
| Q16658 | Fascin | 0.55 |
| P49327 | Fatty acid synthase | 0.55 |
| Q96NE9 | FERM domain-containing protein 6 | 0.43 |
| Q86UX7 | Fermitin family homolog 3 | 0.49 |
| P02794 | Ferritin heavy chain | 1.75 |
| Q14512 | Fibroblast growth factor-binding protein 1 | 1.53 |
| P02751 | Fibronectin | 2.25 |
| O75369 | Filamin-B | 0.19 |
| **O75955** | **Flotillin-1** | 1.89 |
| **Q14254** | **Flotillin-2** | 2.09 |
| Q9NQ84 | G-protein coupled receptor family C group 5 member C | 0.40 |
| P09382 | Galectin-1 | 0.17 |
| P06744 | Glucose-6-phosphate isomerase | 2.12 |
| Q7RTV2 | Glutathione S-transferase A5 | 0.44 |
| P41250 | Glycine--tRNA ligase | MYOF |
| P11216 | Glycogen phosphorylase, brain form | MYOF |
| P35052 | Glypican-1 | 1.64 |
| P49915 | GMP synthase [glutamine-hydrolyzing] | MYOF |
| Q7Z5G4 | Golgin subfamily A member 7 | 0.49 |
| P62826 | GTP-binding nuclear protein Ran | 0.64 |
| P63244 | Guanine nucleotide-binding protein subunit beta-2-like 1 | 0.30 |
| P09651 | Heterogeneous nuclear ribonucleoprotein A1 | 0.47 |
| Q14103 | Heterogeneous nuclear ribonucleoprotein D0 | 2.20 |
| P61978 | Heterogeneous nuclear ribonucleoprotein K | 0.32 |
| Q16778 | Histone H2B type 2-E | 2.48 |
| P62805 | Histone H4 | 2.02 |
| P52292 | Importin subunit alpha-1 | MYOF |
| Q14974 | Importin subunit beta-1 | 0.42 |
| Q13308 | Inactive tyrosine-protein kinase 7 | 2.17 |
| P08648 | Integrin alpha-5 | 0.34 |
| Q9NPH3 | Interleukin-1 receptor accessory protein | MYOF |
| O15554 | Intermediate conductance calcium-activated potassium channel protein 4 | CTRL |
| O75874 | Isocitrate dehydrogenase [NADP] cytoplasmic | 2.76 |
| P41252 | Isoleucine--tRNA ligase, cytoplasmic | 0.29 |
| Q96J84 | Kin of IRRE-like protein 1 | 3.15 |
| O43278 | Kunitz-type protease inhibitor 1 | 0.34 |
| Q13751 | Laminin subunit beta-3 | 0.21 |
| Q13753 | Laminin subunit gamma-2 | 0.53 |
| O43813 | LanC-like protein 1 | MYOF |
| Q08722 | Leukocyte surface antigen CD47 | 0.43 |
| Q7Z4F1 | Low-density lipoprotein receptor-related protein 10 | 1.73 |
| P11279 | Lysosome-associated membrane glycoprotein 1 | 4.56 |
| P40926 | Malate dehydrogenase, mitochondrial | MYOF |
| Q99102 | Mucin-4 | 3.01 |
| P33527 | Multidrug resistance-associated protein 1 | MYOF |
| **Q9NZM1** | **Myoferlin** | 11.68 |
| Q15223 | Nectin-1 | 1.54 |
| Q9BT67 | NEDD4 family-interacting protein 1 | 3.46 |
| Q9NV92 | NEDD4 family-interacting protein 2 | CTRL |
| Q09666 | Neuroblast differentiation-associated protein AHNAK | 0.61 |
| Q14697 | Neutral alpha-glucosidase AB | 0.66 |
| Q96TA1 | Niban-like protein 1 | MYOF |
| P43490 | Nicotinamide phosphoribosyltransferase | MYOF |
| O15118 | Niemann-Pick C1 protein | 5.71 |
| P06748 | Nucleophosmin | 0.30 |
| Q9NTK5 | Obg-like ATPase 1 | CTRL |
| Q99650 | Oncostatin-M-specific receptor subunit beta | 1.68 |
| Q9BTU6 | Phosphatidylinositol 4-kinase type 2-alpha | CTRL |
| P36969 | Phospholipid hydroperoxide glutathione peroxidase, mitochondrial | MYOF |
| Q99569 | Plakophilin-4 | 0.41 |
| P05120 | Plasminogen activator inhibitor 2 | 0.16 |
| P13797 | Plastin-3 | 0.32 |
| Q15149 | Plectin | 0.59 |
| Q9UKK3 | Poly [ADP-ribose] polymerase 4 | 2.05 |
| Q15365 | Poly(rC)-binding protein 1 | 0.63 |
| Q15366 | Poly(rC)-binding protein 2 | MYOF |
| P11940 | Polyadenylate-binding protein 1 | 0.63 |
| P09668 | Pro-cathepsin H | 0.44 |
| Q9H3G5 | Probable serine carboxypeptidase CPVL | CTRL |
| Q02809 | Procollagen-lysine,2-oxoglutarate 5-dioxygenase 1 | MYOF |
| P12004 | Proliferating cell nuclear antigen | MYOF |
| Q9UQ80 | Proliferation-associated protein 2G4 | 0.14 |
| P28072 | Proteasome subunit beta type-6 | 2.55 |
| Q99873 | Protein arginine N-methyltransferase 1 | 0.43 |
| P07237 | Protein disulfide-isomerase | 1.60 |
| Q92520 | Protein FAM3C | 1.60 |
| Q92597 | Protein NDRG1 | 0.39 |
| Q9UGV2 | Protein NDRG3 | MYOF |
| P60903 | Protein S100-A10 | 4.45 |
| Q9C0H2 | Protein tweety homolog 3 | 1.59 |
| Q9GZT5 | Protein Wnt-10a | CTRL |
| O75695 | Protein XRP2 | 1.58 |
| Q14517 | Protocadherin Fat 1 | 0.49 |
| Q9UN70 | Protocadherin gamma-C3 | 1.52 |
| O43865 | Putative adenosylhomocysteinase 2 | MYOF |
| O43143 | Putative pre-mRNA-splicing factor ATP-dependent RNA helicase DHX15 | MYOF |
| P14618 | Pyruvate kinase PKM | 0.50 |
| P35241 | Radixin | 2.10 |
| P46940 | Ras GTPase-activating-like protein IQGAP1 | 0.55 |
| P51149 | Ras-related protein Rab-7a | 1.59 |
| Q9Y3L5 | Ras-related protein Rap-2c | 0.46 |
| P10586 | Receptor-type tyrosine-protein phosphatase F | 1.54 |
| O14828 | Secretory carrier-associated membrane protein 3 | 2.25 |
| Q12884 | Seprase | CTRL |
| Q13501 | Sequestosome-1 | 2.51 |
| O15269 | Serine palmitoyltransferase 1 | 1.52 |
| Q92743 | Serine protease HTRA1 | 3.01 |
| Q7KZI7 | Serine/threonine-protein kinase MARK2 | MYOF |
| P30153 | Serine/threonine-protein phosphatase 2A 65 kDa regulatory subunit A alpha isoform | 0.54 |
| P62318 | Small nuclear ribonucleoprotein Sm D3 | MYOF |
| Q8NCG7 | Sn1-specific diacylglycerol lipase beta | CTRL |
| Q96QD8 | Sodium-coupled neutral amino acid transporter 2 | 0.52 |
| Q08357 | Sodium-dependent phosphate transporter 2 | 2.11 |
| P19634 | Sodium/hydrogen exchanger 1 | MYOF |
| P05026 | Sodium/potassium-transporting ATPase subunit beta-1 | 0.45 |
| P54709 | Sodium/potassium-transporting ATPase subunit beta-3 | 2.25 |
| Q9UMY4 | Sorting nexin-12 | 2.37 |
| Q15036 | Sorting nexin-17 | CTRL |
| P38646 | Stress-70 protein, mitochondrial | MYOF |
| Q8TED4 | Sugar phosphate exchanger 2 | CTRL |
| O43760 | Synaptogyrin-2 | 1.65 |
| O43752 | Syntaxin-6 | 1.79 |
| P17987 | T-complex protein 1 subunit alpha | 0.60 |
| P78371 | T-complex protein 1 subunit beta | 0.60 |
| P50991 | T-complex protein 1 subunit delta | 0.55 |
| P48643 | T-complex protein 1 subunit epsilon | 0.44 |
| Q99832 | T-complex protein 1 subunit eta | 0.60 |
| P50990 | T-complex protein 1 subunit theta | 0.67 |
| P40227 | T-complex protein 1 subunit zeta | 0.65 |
| Q86VP1 | Tax1-binding protein 1 | 2.09 |
| O95858 | Tetraspanin-15 | CTRL |
| P26639 | Threonine--tRNA ligase, cytoplasmic | 0.46 |
| P07204 | Thrombomodulin | 0.36 |
| P00750 | Tissue-type plasminogen activator | 3.70 |
| P02786 | Transferrin receptor protein 1 | 1.69 |
| Q15582 | Transforming growth factor-beta-induced protein ig-h3 | 0.10 |
| P29401 | Transketolase | 1.87 |
| Q7Z403 | Transmembrane channel-like protein 6 | 0.42 |
| Q9BXS4 | Transmembrane protein 59 | 1.51 |
| P29144 | Tripeptidyl-peptidase 2 | 2.95 |
| P07437 | Tubulin beta chain | 0.66 |
| Q14166 | Tubulin--tyrosine ligase-like protein 12 | MYOF |
| P21580 | Tumor necrosis factor alpha-induced protein 3 | 0.59 |
| O75509 | Tumor necrosis factor receptor superfamily member 21 | 2.13 |
| P23458 | Tyrosine-protein kinase JAK1 | 2.10 |
| O75643 | U5 small nuclear ribonucleoprotein 200 kDa helicase | MYOF |
| P61088 | Ubiquitin-conjugating enzyme E2 N | 0.37 |
| Q8IX04 | Ubiquitin-conjugating enzyme E2 variant 3 | 0.64 |
| P22314 | Ubiquitin-like modifier-activating enzyme 1 | 0.53 |
| Q9P206 | Uncharacterized protein KIAA1522 | MYOF |
| Q16851 | UTP--glucose-1-phosphate uridylyltransferase | 0.53 |
| O75436 | Vacuolar protein sorting-associated protein 26A | MYOF |
| A5D8V6 | Vacuolar protein sorting-associated protein 37C | 2.25 |
| P50552 | Vasodilator-stimulated phosphoprotein | 2.03 |
| Q9UEU0 | Vesicle transport through interaction with t-SNAREs homolog 1B | 2.59 |
| P51809 | Vesicle-associated membrane protein 7 | 1.88 |
| O75083 | WD repeat-containing protein 1 | 0.52 |
| Q9Y6W5 | Wiskott-Aldrich syndrome protein family member 2 | MYOF |
| P13010 | X-ray repair cross-complementing protein 5 | MYOF |
