# Supplementary material for: Myoferlin is a novel exosomal protein and functional regulator of cancer-derived exosomes
Source: Oncotarget. 2016 Nov 10;7(50):83669–83. doi: 10.18632/oncotarget.13276 (PMC5347796; doi:10.18632/oncotarget.13276)
Supplement: Supplementary file 4 [file oncotarget-07-83669-s004.docx]

**Supplementary Table S3**

**Proteins down-regulated in common (MDA-MB-231 and BxPC3 cells) following myoferlin silencing.**

| **Accession** | **Protein Description** | **CTRL/MYOF Ratio MDA-MB-231** | **CTRL/MYOF Ratio BXPC-3** |
| --- | --- | --- | --- |
| P62191 | 26S protease regulatory subunit 4 | CTRL | CTRL |
| P11021 | 78 kDa glucose-regulated protein | 2.56 | 3.66 |
| Q9NVJ2 | ADP-ribosylation factor-like protein 8B | 8.22 | 1.85 |
| O43707 | Alpha-actinin-4 | 1.51 | 1.98 |
| Q03135 | Caveolin-1 | 2.17 | 1.77 |
| P08962 | CD63 antigen | CTRL | 2.29 |
| P02794 | Ferritin heavy chain | 2.63 | 1.75 |
| O75955 | Flotillin-1 | 4.22 | 1.89 |
| Q14254 | Flotillin-2 | 4.25 | 2.09 |
| P62805 | Histone H4 | 1.88 | 2.02 |
| O75874 | Isocitrate dehydrogenase [NADP] cytoplasmic | CTRL | 2.76 |
| Q7Z4F1 | Low-density lipoprotein receptor-related protein 10 | CTRL | 1.73 |
| Q9NZM1 | Myoferlin | CTRL | 11.68 |
| O15118 | Niemann-Pick C1 protein | 3.85 | 5.71 |
| Q99650 | Oncostatin-M-specific receptor subunit beta | CTRL | 1.68 |
| Q9BTU6 | Phosphatidylinositol 4-kinase type 2-alpha | CTRL | CTRL |
| Q9UKK3 | Poly [ADP-ribose] polymerase 4 | 4.87 | 2.05 |
| P07237 | Protein disulfide-isomerase | CTRL | 1.60 |
| Q9C0H2 | Protein tweety homolog 3 | 2.15 | 1.59 |
| P51149 | Ras-related protein Rab-7a | 1.55 | 1.59 |
| P10586 | Receptor-type tyrosine-protein phosphatase F | 3.97 | 1.54 |
| O14828 | Secretory carrier-associated membrane protein 3 | 3.23 | 2.25 |
| Q13501 | Sequestosome-1 | CTRL | 2.51 |
| Q8NCG7 | Sn1-specific diacylglycerol lipase beta | CTRL | CTRL |
| P54709 | Sodium/potassium-transporting ATPase subunit beta-3 | CTRL | 2.25 |
| Q15036 | Sorting nexin-17 | CTRL | CTRL |
| O43752 | Syntaxin-6 | CTRL | 1.79 |
| Q86VP1 | Tax1-binding protein 1 | CTRL | 2.09 |
| P02786 | Transferrin receptor protein 1 | 3.02 | 1.69 |
| P29144 | Tripeptidyl-peptidase 2 | 2.77 | 2.95 |
| P23458 | Tyrosine-protein kinase JAK1 | CTRL | 2.10 |
| P51809 | Vesicle-associated membrane protein 7 | CTRL | 1.88 |

**Proteins up-regulated in common (MDA-MB-231 and BxPC3 cells) following myoferlin silencing.**

| **Accession** | **Protein Description** | **CTRL/MYOF Ratio MDA-MB-231** | **CTRL/MYOF Ratio BXPC-3** |
| --- | --- | --- | --- |
| P61769 | Beta-2-microglobulin | 0.55 | 0.20 |
| Q7RTV2 | Glutathione S-transferase A5 | 0.54 | 0.44 |
| Q96TA1 | Niban-like protein 1 | MYOF | MYOF |
| Q15582 | Transforming growth factor-beta-induced protein ig-h3 | 0.36 | 0.10 |
